# Colonic Submucosa Targeted Delivery of Probiotic and Rhein for Ulcerative Colitis Treatment

**DOI:** 10.1002/advs.202409711

**Published:** 2025-05-09

**Authors:** Lingqiang Li, Linxin Dai, Meisi Lin, Shuang He, Hongye Du, Dasheng Lin, Yanbin Wang, Fenglian Zhang, Sian Tao, Xiaoluo Sun, Xinggui Huang, Haihui Liu, Qian Wang, Lingling He, Kunhe Wu, Jieshu You, Minyue Zhang, Chaomei Fu, He Tu, Naijing Ye, Jibin Liu, Fei Gao

**Affiliations:** ^1^ State Key Laboratory of Southwestern Chinese Medicine Resources School of Pharmacy / School of Modern Chinese Medicine Industry Chengdu University of Traditional Chinese Medicine Chengdu 611130 China; ^2^ College of Basic Medical Sciences Chengdu University of Traditional Chinese Medicine Chengdu 611130 China; ^3^ TCM Regulating Metabolic Diseases Key Laboratory of Sichuan Province Hospital of Chengdu University of Traditional Chinese Medicine Chengdu 610072 China; ^4^ Chengdu Huashen Technology Group Co., Ltd. Chengdu 611137 China; ^5^ College of Pharmacy Shenzhen Technology University Shenzhen Guangdong 518118 China; ^6^ Division of Hematology of Renji Hospital School of Medicine Shanghai Jiaotong University Shanghai 200127 China

**Keywords:** bacillus subtilis, rhein, submucosal drug delivery system, ulcerative colitis, yeast cell wall microparticles

## Abstract

Ulcerative colitis (UC) is a chronic disease. A significant challenge for the management of UC is to achieve delivery of drugs to the multi‐layer colonic barriers, as existing drugs are difficult to penetrate these depths. In this study, a novel drug delivery system using yeast cell wall microparticles (YPs) are developed to co‐encapsulate *Bacillus subtilis* (BS) and Rhein (Rh) termed Rh‐YBS. This system specifically targets colonic microfold cells, enabling direct delivery of BS to the colonic submucosa. Additionally, Rh enhances BS colonization in the submucosa through floral regulation. Studies indicate that Rh‐YBS can effectively reach and proliferate within the submucosa in vivo. In a DSS‐induced UC mouse model, Rh‐YBS stimulates the CGRP‐related neural pathway; BS activation in the submucosa leads to increased CGRP secretion, prompting goblet cells to secrete mucus and thereby repairing the mucosa. Furthermore, the Rh‐YBS also provide a preventive benefit against UC. In summary, Rh‐YBS represents an innovative drug delivery system for mucosal repair in UC treatment, activating a unique mechanism involving the CGRP‐related neural pathway.

## Graphical abstract

A novel orally drug‐probiotic co‐delivery system designated as Rh‐YBS for UC treatment, which highlights three innovations: (i) YPs involves colonic submucosa delivery via M cells; (ii) Rh (drug) promotes the colonization and growth of BS (probiotic) in the colon. (iii) BS induces the CGRP (a novel candidate target) secretion to inhibit colonic inflammatory response.

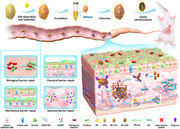

## 1 Introduction

Ulcerative colitis (UC) is an inflammatory disease of the colon characterized by symptoms such as diarrhea, bloody mucus, and recurrent abdominal pain.^[^ ^1^, ^2^, ^3^, ^4^, ^5^, ^6^ ^]^ In severe instances, it may result in significant hemorrhage, toxic megacolon, or other complications.^[^ ^7^, ^8^, ^9^ ^]^ Previous studies have shown that UC patients have a 2–3 times greater risk of developing colorectal cancer than healthy individuals.^[^ ^10^, ^11^, ^12^, ^13^ ^]^ The global prevalence of UC was estimated to reach 5 million cases in 2023.^[^ ^14^, ^15^, ^16^ ^]^ Currently, the main clinical drugs for UC treatment are immunosuppressants such as 5‐aminosalicylic acid and glucocorticoids, which can offer symptom relief but are limited by their singular therapeutic approach and notable side effects.^[^ ^17^, ^18^, ^19^ ^]^ The primary objective of UC treatment is to restore the integrity of colonic barriers, including biological, chemical, mechanical, and immune barriers, which relies on multi‐layer intervention.^[^ ^20^, ^21^ ^]^ Nowadays, most of extent drug delivery systems could only reach in the superficial layer of the colon (where the biological, chemical, and mechanical barriers reside),^[^ ^22^ ^]^ the submucosa—where immune barriers, nerves, blood vessels, and so on reside, lies deeper within the colon, making it difficult to reach. This limitation severely restricts the effectiveness of multi‐layer intervention. Furthermore, certain mechanisms associated with submucosa in UC treatment, such as immune hyperactivity,^[^ ^23^ ^]^ bacterial invasion,^[^ ^24^, ^25^ ^]^ and the brain‐gut axis,^[^ ^26^ ^]^ are infeasible due to the absence of an appropriate drug delivery system. Consequently, overcoming the structural barriers of the colon to access the submucosa has emerged as a critical challenge in the treatment of UC.^[^ ^27^ ^]^ This study aims to construct a targeted drug delivery system for the colon submucosa to address this significant issue.

Microfold cells (M cells), which serve as components of the mechanical barrier and act as a “gateway” to the immune barrier, are potential targets for drug delivery to the submucosa.^[^ ^28^, ^29^ ^]^ M cells facilitates the passage of macromolecular substances into the deeper layers of the colon. To target M cells, the yeast cell wall microparticles (YPs) were selected for research. YPs are natural polysaccharide components that have been extensively studied.^[^ ^30^, ^31^ ^]^ Furthermore, a novel mechanism in the submucosa has been identified as a promising strategy for UC treatment.^[^ ^26^ ^]^ Specifically, the calcitonin gene‐related peptide (CGRP) related nerve pathway (CGRP‐RN) can be stimulated by intestinal flora to produce CGRP in the submucosa,^[^ ^26^ ^]^ subsequently activates goblet cells to secrete mucus, thereby facilitating the repair of the colonic mucosa. *Bacillus subtilis* (BS) was widely used in UC treatment,^[^ ^32^, ^33^, ^34^ ^]^ which could stimulate CGRP‐RN, presenting a prospective mechanism for mucosal repair. However, the application of BS encounters two challenges: first, the method of orally delivering BS to the submucosa remains unclear. YPs, previously mentioned, can target M cell due to their gastric stability and β‐glucan content. Second, supporting BS survival and colonization is crucial. YPs could provide nutrition for BS proliferation.^[^ ^35^ ^]^ In our previous experiments, ingredients of rhubarb had been studied,^[^ ^36^, ^37^, ^38^, ^39^, ^40^ ^]^ the Rhein (Rh) is effective in regulating the colonic flora, aiding BS colonization by inhibiting pathogenic bacteria.

**Scheme 1:**
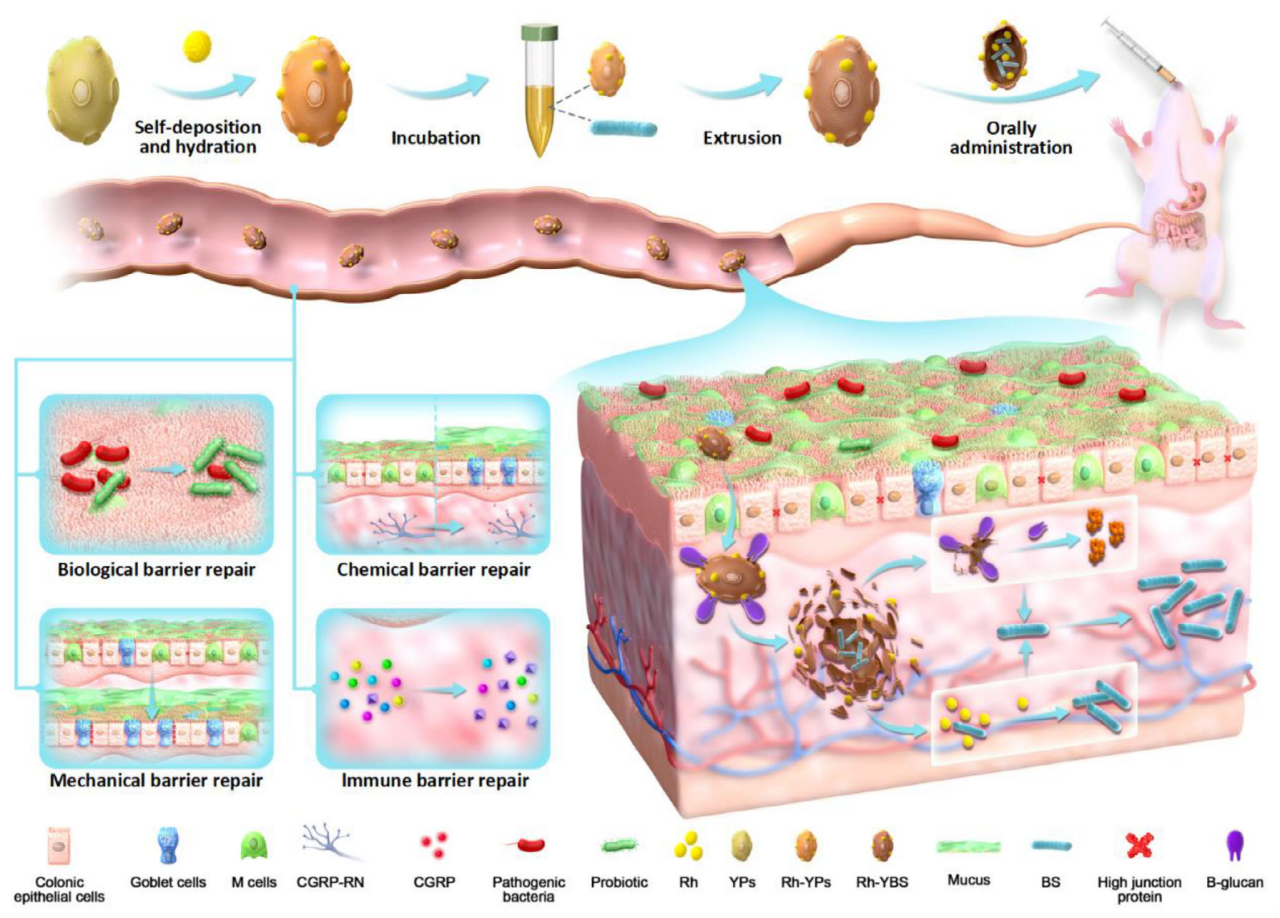
Schematic illustration showing the preparation of Rh‐YPs and the progress of Rh‐YPs treat UC via CGRP‐RN pathway.

In this study, Rh and YPs were combined by electrostatic force‐driven self‐deposition and solvent hydration/lyophilization method, which encapsulates BS, forming a novel oral drug delivery system Rh‐YBS. We characterized this delivery system and evaluated its therapeutic effects on UC, confirmed the contributions of BS, YPs and Rh. Notably, Rh‐YBS enhanced BS delivery to the colonic submucosa, and activated the CGRP‐RN pathway. Overall, we designed a new drug delivery system aimed at targeting the colon submucosa for the treatment of UC (**Scheme** **1**).

## 2 Results and Discussion

### 2.1 Preparation and Characterization

To prepare Rh‐YBS, Rh was combined with YPs to form Rh‐YPs, followed by encapsulation of BS, resulting in the product Rh‐YBS. The form and structure of Rh‐YBS was identified by ultraviolet spectrum (UV), infrared spectroscopy (IR), and electron microscope etc.

**Figure 1:**
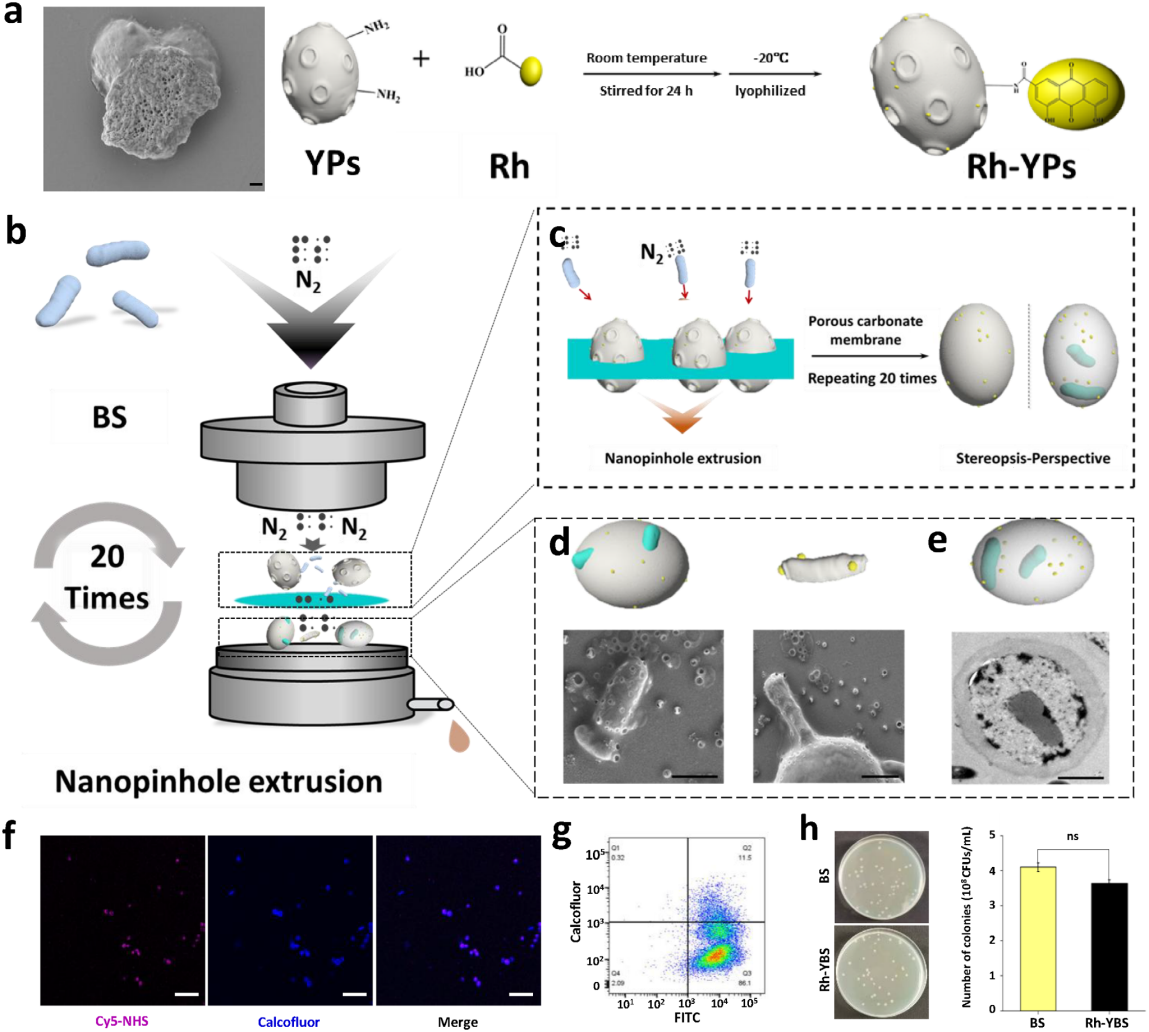
Preparation and characterization of Rh‐YBS. a) SEM image of YPs and Rh‐YPs, and schematic illustration of probable combination form about Rh and YPs. Scale bar: 1 µm. b) and c) Schematic illustration of extrusion method. d) Schematic illustration and SEM images of Rh‐YBS. Scale bar: 1 µm. e) Schematic illustration and TEM images of Rh‐YBS. Scale bar: 1 µm. f). LSCM images of Rh‐YBS. Cy5‐NHS represented BS, Calcofluor represented YPs. Scale bars: 20 µm. g). FCW results of Rh‐YBS. YPs was stain by Calcofluor, BS was stain by FITC‐NHS h) Colonies number of BS and Rh‐YBS on LB plate.

Initially, Rh and YPs were combined by electrostatic force‐driven self‐deposition and solvent hydration/lyophilization.^[^ ^23^, ^24^ ^]^ Specifically, Rh was dissolved in dimethyl sulfoxide, then mixed with YPs, incubated at room temperature for 24 h. After dialyzing the reaction solution, the precipitate was collected and freeze‐dried to obtain Rh‐YPs (Figure S1, Supporting Information). The characterization methods employed were the same as our previous research.^[^ ^31^ ^]^ UV and IR were used for further information about Rh‐YPs.^[^ ^41^ ^]^ UV analysis revealed characteristic absorption peaks of Rh (500 nm) and YPs (250 nm), confirming the presence of both components in the Rh‐YPs (Figure S2a, Supporting Information). Results. of IR was shown in Figure S2b (Supporting Information). It was found that, peak of Rh disappeared, which mean Rh was combined with YPs after synthetization. Moreover, an enhanced infrared absorption from 750 cm^−1^ to 400 cm^−1^ was noted in the Rh‐YPs sample, which mean that the combination of Rh and YPs was formed partly through amido bond (**Figure** **1**a). Additionally, the content of Rh was determined by high‐performance liquid chromatography,^[^ ^42^ ^]^ encapsulation rate of Rh at approximately 53.5%, and a Rh content of 0.27 mg per 1 mg of YPs (Table S1, Supporting Information). These results confirmed the successful preparation of Rh‐YPs.

Rh‐YBS were prepared using a modified extrusion method (Figure 1b).^[^ ^43^ ^]^ BS was cultured in Luria‐Bertani (LB) liquid medium for 6 h, achieving a concentration of 10^8^ CFU/mL, BS was then mixed with Rh‐YPs and passed through a small extruder (Figure 1c). Following preparation, samples were examined under a scanning electron microscope (SEM), which revealed a smooth surface on BS and lumpy protrusions distributed on Rh‐YPs (Figure S3, Supporting Information). The presence of these lumpy protrusions on Rh‐YBS after extrusion was noted (Figure 1d), indicating that BS was effectively covered by Rh‐YPs. Further microscopic analysis using a transmission electron microscope (TEM) compared to earlier images of BS and Rh‐YPs (Figure S4, Supporting Information), showing BS visibly located within the pores of Rh‐YBS (Figure 1e). SEM and TEM analyses indicated that Rh‐YBS encapsulation of BS occurred in three forms: fully covered, partially encapsulated, and completely encapsulated (Figure 1e,f). For an overall visualization, confocal laser scanning microscope (CLSM) was employed. It was observed that Rh‐YPs (blue area) were densely populated by BS (pink area), illustrating the successful formation of Rh‐YBS (Figure 1f).

The encapsulation efficiency of BS was determined by flow cytometry (FCW). BS was stained with FITC‐NHS, while Rh‐YPs were stained with calcofluor. Results showed that approximately 11.7% of BS was coated by Rh‐YPs (Q2), with the total BS population accounting for nearly 97.0% (Q2 + Q3), yielding an encapsulation efficiency of 12.1% [Q2/ (Q2 + Q3)] (Figure 1g).

The survival rate of BS was assessed using the plate counting method. BS samples before and after preparation were incubated on LB solid medium for 12 h, the colonies numbers of BS were counted. The results shown that no significant difference was observed before and after preparation (*p* > 0.05) (Figure 1h), indicating that the extrusion method had no adverse effect on the biological activity of BS. Through a series of preparation and characterization steps, Rh‐YBS was successfully produced for the further studies.

### 2.2 Contributions of YPs to Rh‐YBS

YPs play a crucial role in protecting BS against gastric acids and promoting the proliferation of BS after releasing,^[^ ^24^, ^28^, ^29^, ^34^ ^]^ which are essential for the oral delivery of Rh‐YBS. The following experiments were conducted to validate the contributions of YPs.

**Figure 2:**
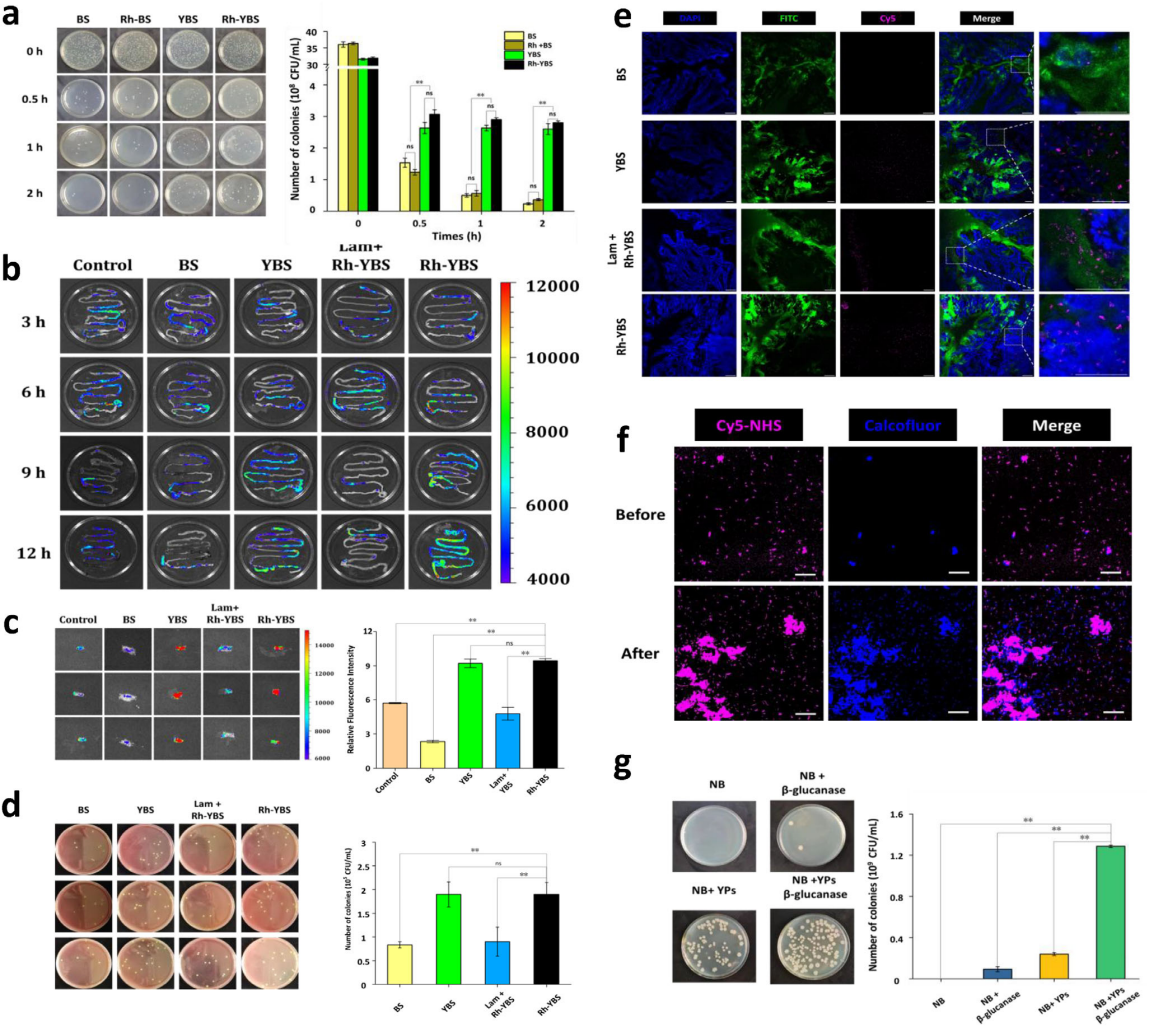
Contributions of YPs to Rh‐YBS. a) Number of colonies of BS in different groups after SGF treatment (n = 3). b) The colon fluorescence images of mice with orally treated by different therapeutic formulas at different time point. c) The colon fluorescence images and histogram of colons treated soaked in different therapeutic formulas (n = 3). d) The colonize counts and histogram of BS across through the M cells(n = 3). e) Frozen sections of colon tissue. The blue area represented colon tissue, green area represented mucosa, pink area represented BS. Scale bar: 50 µm. f) CLSM images of Rh‐YBS treated with or without β‐glucanase. Scale bar: 50 µm. g) The colonized counts of BS after 12 h incubated in different formulas (n = 3). Data are mean ± SE. *p < 0.05; **p < 0.01.

The gastric stability of Rh‐YBS was tested using simulated gastric fluid (SGF, pH = 1.2). Samples were exposed to SGF for 2 h, then cultured on LB solid medium. The number of colonies of BS was shown in **Figure** **2**a. Compared BS versus Rh‐BS groups and YBS versus Rh‐YBS groups at the 0.5_th_, 1_st_ and 2_nd_ h, it was found that Rh could not protect BS against SGF (*p* > 0.05). However, the number of colonies of Rh‐YBS and YBS groups were obviously higher than BS and Rh‐BS groups (*p* < 0.01), indicating that YPs provide a protective effect on BS against SGF. SEM analysis before and after SGF exposure showed that BS was damaged by SGF, while Rh‐YBS maintained intact structure (Figure S5, Supporting Information). These results demonstrate that Rh‐YBS has gastric stability due to the presence of YPs.

The colon targeting and retention abilities of Rh‐YBS, derived from YPs, were assessed by the following experiments. Initially, BS was labeled with Cy5‐NHS, and the distribution of different formulations in the digestive tract was analyzed using an in vivo imaging system (IVIS), laminarin (Lam) was used to block down M cell to confirm if Rh‐YBS can target M cells.^[^ ^44^, ^45^, ^46^ ^]^ Mice treated by DSS were orally administered with BS, Rh‐YBS, and Lam + Rh‐YBS. Healthy mice orally administrated with Rh‐YBS was set as Control group, aiming to learn if Rh‐YBS could be retained in a healthy colon. Then, mice in each group were sacrificed at the 3_rd_, 6_th_, 9_th_, and 12_th_ h post‐administration. The digestive tract below the stomach of mice were removed and imaged. Figure 2b shown images of each group. Compared to the BS group, the YBS and Rh‐YBS groups exhibited higher fluorescence in the colon at the 9_th_ and 12_th_ h after administration, indicating that YPs could prolong the retention time of BS in a colitis colon. Meanwhile, the fluorescence of the Control and Lam + Rh‐YBS groups was decreased instantly, suggesting that the colon retention ability of Rh‐YBS may related with M cell. Subsequently, a tissue‐level study was conducted. Mice were sacrificed, and their colons were harvested for evaluation. After treatment, the colons were examined under an IVIS. The images and statistical histograms of fluorescence intensity are displayed in Figure 2c. It was found that the fluorescence intensity in the YBS and Rh‐YBS groups were significantly higher than that in the Control and BS groups (*p* < 0.01), and decreased significantly under the influence of Lam (*p* < 0.01, compared with the Rh‐YBS group). This result indicates that YPs could enhance the retention capacity of BS in colon tissue, potentially related to M cells. Furthermore, to verify the effect of Rh‐YBS in targeting M cells at the cellular level, M cells were cultured with different preparations in vitro. The medium from the basolateral sides of trans‐well inserts was collected and plated onto Bacilus Cereus Selective solid medium for BS quantification. It was observed that the number of BS crossing M cells in YBS and Rh‐YBS groups was significantly higher than that of BS and Lam + Rh‐YBS groups (*p* < 0.01). The results proved that BS could across the M cells with the assistance of YPs (Figure 2d). These experiments demonstrated that Rh‐YBS could enhance the retention of BS in the colon through M cells. Lastly, to confirm whether BS remains in the submucosa due to M cell uptake, different preparations were orally administered to mice, which were then sacrificed after about 12 h. The colons were harvested, sectioned, and stained for observation under a CLSM. It was shown that BS (pink area) in both the YBS and Rh‐YBS groups was distributed in both the mucosa (green area) and submucosa (blue area). However, BS in the Lam + Rh‐YBS group and the BS group could only be found in the mucosa (Figure 2e). These results demonstrate that YPs can assist BS in reaching the submucosa through M cells. These results suggest that Rh‐YBS has the ability to target M cells and retain in the colon, owing to the contributions of YPs.

To investigate the release mechanism of Rh‐YBS in the submucosal microenvironment, an in vitro model simulating enzymatic degradation by colonic microbiota was developed. Rh‐YBS complexes were incubated with β‐glucanase, microbial enzyme that catalyzes the breakdown of β‐glucans in YPs,^[^ ^27^, ^43^ ^]^ for 2 h. CLSM analysis revealed the structural disintegration of YPs and subsequent release of BS following enzymatic treatment (Figure 2f). This controlled degradation process confirms the enzymatic responsiveness of the delivery system and validates the proposed release mechanism in the colonic environment. To confirm the capability of YPs to promote the proliferation of BS, modified Chu/Gamborg basal (NB) medium with various formulations was used to amplify BS. As depicted in Figure 2g, the count of BS on the LB agar plate for the NB + YPs + β‐glucanase group was significantly higher than that of the other groups (*p* < 0.01). These results demonstrated that YPs could enhance the proliferation of BS.

The experiments described above provide substantial evidence for the contributions of YPs. Furthermore, they elucidate the in vivo distribution process of Rh‐YBS: traveling through the stomach protected by YPs, targeting M cells to enter the submucosa around the colon, and proliferating with the assistance of YPs metabolites in the submucosa. This process lays the groundwork for subsequent experiments.

### 2.3 The Effects of Rh‐YBS In Vivo

In the above experiments, Rh‐YBS was successfully synthesized and demonstrated the potential for oral administration, benefiting from the protective effect of YPs. Therefore, the therapeutic efficacy of Rh‐YBS on UC was evaluated through in vivo experiments.

**Figure 3:**
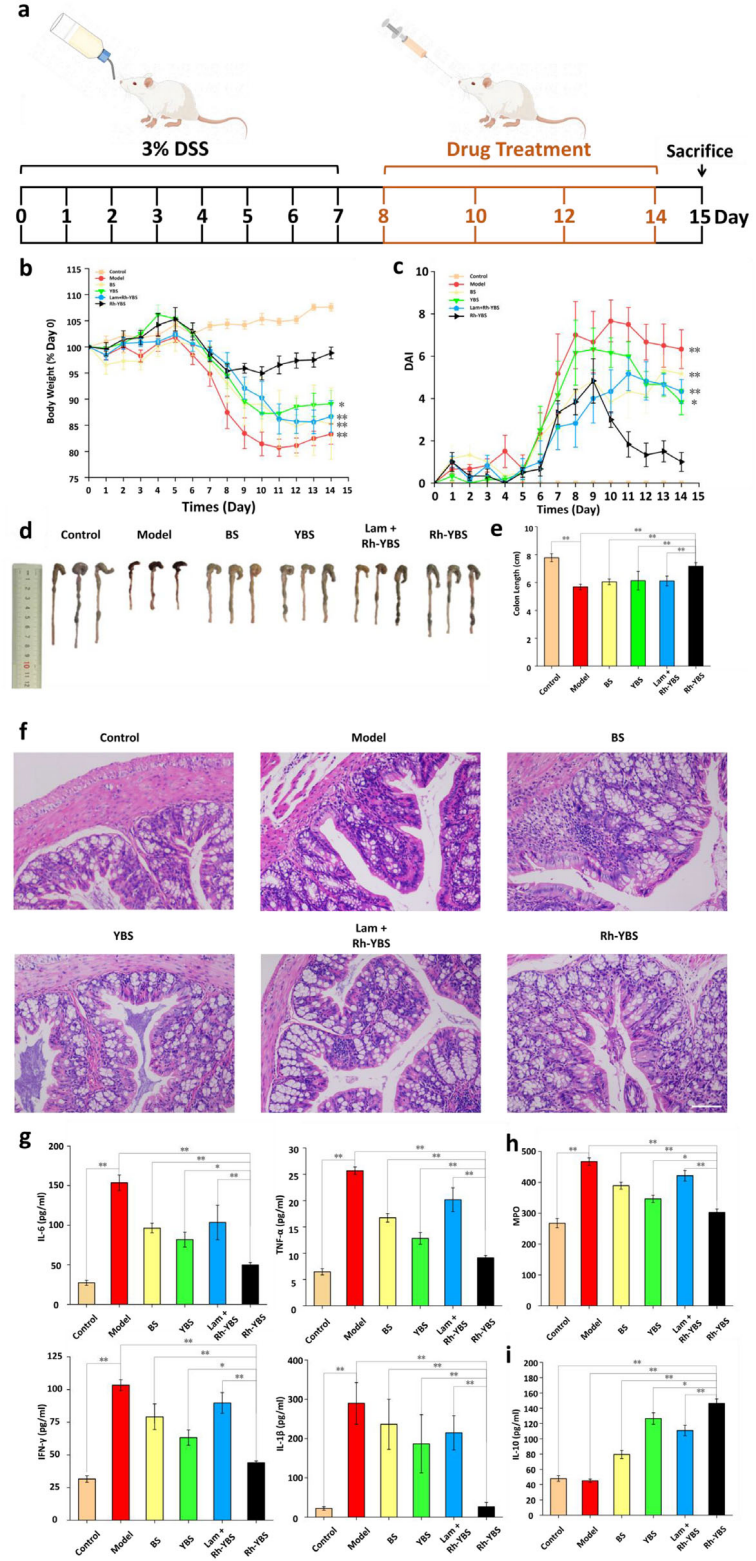
The effects of Rh‐YBS in vivo. a) Therapeutic procedure of different therapeutic formulations to DSS mice. b) Broken‐line graph of body weight varying percent (compared to day 0) with time among different therapeutic formulas (n = 6). c) Broken‐line graph of DAI scores varying with time among different groups (n = 6). d) Images of colons among different groups. e) Histogram of colons length among different groups (n = 6). f) H&E staining of colonic sections among different groups. Scale bars: 100 µm. g)–i) Histogram analysis of different groups on the levels of (g) inflammatory cytokines (IL‐6, TNF‐α, IFN‐γ, and IL‐1β), (h) oxidation cytokine (MPO), (i) anti‐inflammatory cytokine (IL‐10) in the colon (n = 6). Data are mean ± SE. *p < 0.05; **p < 0.01.

DSS‐induced mice were administered with various therapeutic formulations via oral gavage on the 8_th_, 10_th_, 12_th_, and 14_th_ days following DSS administration and were sacrificed on the 15_th_ day (**Figure** **3**a). The body weight, incidence of diarrhea, and severity of bloody stools were recorded daily. As shown in Figure 3b, the body weight of the mice consistently decreased from the 5_th_ day after DSS administration. After treatment with Rh‐YBS, there was an improvement in the body weight from the 8_th_ day. Moreover, by the endpoint, the body weight of mice in the Rh‐YBS group was similar to that of the Control group, significantly higher than the other groups (*p* < 0.05 compared to the YBS group, *p* < 0.01 compared to other groups). Figure 3c illustrates that mice treated with DSS exhibited severe enteritis symptoms starting from the 6_th_ day. After Rh‐YBS treatment, symptoms began to alleviate on the 9_th_ day. The symptoms of enteritis in the Rh‐YBS group were significantly milder than those in other DSS‐treated groups on the 14_th_ day (*p* < 0.05 compared to the YBS group, *p* < 0.01 compared to other groups). Figure 3d,e showed that the colon length in the Model group was significantly shortened compared to the Control group (*p* < 0.01), but it recovered after being treated with therapeutic formulations. Moreover, the colon length in the Rh‐YBS group was significantly longer than that in other therapeutic groups (*p* < 0.01). These results indicate that Rh‐YBS has a therapeutic effect on UC, and this effect is stronger than that of BS or YBS alone. Importantly, across all measured indices, the Rh‐YBS group exhibited a significantly better effect than the Lam + Rh‐YBS group (*p* < 0.01), indicating a potential correlation between M cells and the therapeutic efficacy of Rh‐YBS against UC, which is consistent with the conclusions drawn from prior studies.

Hematoxylin‐eosin staining (H&E) was employed to confirm the therapeutic effect of Rh‐YBS on UC and assess the toxicity of Rh‐YBS on organs. As depicted in Figure 3f, the Model group exhibited severe mucosal damage characterized by focal infiltration of inflammatory cells, crypt loss, and necrosis within the colonic tissues, compared to the Control group. The colons from the Rh‐YBS group showed more intact crypts and tissue, indicating that Rh‐YBS ameliorated the signs and symptoms of colonic inflammation. Additionally, images of the heart, liver, spleen, lung, and kidney from each group showed no histopathological evidence of damage (Figure S6, Supporting Information). These findings indicate the therapeutic effect of Rh‐YBS on UC biosafety on organs.

The levels of cytokines were assessed to confirm the efficacy of Rh‐YBS in UC treatment using an enzyme‐linked immunosorbent assay (ELISA). Levels of four inflammatory cytokines (IL‐6, TNF‐α, IFN‐γ, and IL‐1β) are presented in Figure 3g. All factors were significantly increased in the Model group compared to the Control group (*p* < 0.01), while Rh‐YBS treatment effectively reduced the levels of these cytokines, which were significantly lower than those seen with other therapeutic formulations (*p* < 0.05). Figure 3h showed that Rh‐YBS could enhance the secretion of anti‐inflammatory factors such as IL‐10, more effectively than other therapeutic formulations (*p* < 0.01). Additionally, the colonic MPO activity in the Model group was significantly higher than in the Control group (*p* < 0.01), and the Rh‐YBS group showed the most significant reduction in MPO levels compared to other preparation groups (*p* < 0.05 compared to the YBS group, *p* < 0.01 compared to other groups) (Figure 3i). Similar results were observed for all six factors in serum, as shown in Figure S7 (Supporting Information). These findings demonstrate that Rh‐YBS plays a pivotal role in mediating anti‐inflammatory responses associated with UC.

Disordered intestinal flora is an important indicator of UC.^[^ ^47^, ^48^ ^]^ To gain further insight, flora sequencing analysis using feces was conducted. As depicted in Figure S8a (Supporting Information), flora diversity in the feces of the Model group decreased compared to the Control group and increased following treatment with various therapeutic formulations. Figure S8b (Supporting Information) shows that the flora diversity in the Rh‐YBS group was closest to the Control group. The family‐level square stacking diagram in Figure S8c (Supporting Information) indicates that the population of *Bacteroidaceae*.^[^ ^49^ ^]^ surged after DSS treatment and decreased following therapeutic interventions. Although the flora composition of the submucosa differs from that of the feces, similar trends in the growth and decrease of *Bacteroidaceae* were observed. A heatmap detailing the relative abundance of submucosa species shows variations at the species level among the groups (Figure S8b, Supporting Information). The relative species abundance of certain bacteria with notable differences is illustrated in a histogram (Figure S8c–f, Supporting Information),^[^ ^50^ ^]^ Specially, *Reuteri* is an acknowledged probiotic used to alleviate UC by regulating immune responses,^[^ ^51^, ^52^, ^53^ ^]^ *Muciniphila* is associated with certain intestinal diseases because of its damage to mucosa,^[^ ^54^ ^]^ *Gordonii* is a symbiotic bacteria that resides in the oral cavity, however, it has the potential to cause inflammation if appeared in inappropriate locations,^[^ ^55^, ^56^ ^]^ *Ovatus* could improve UC by metabolize dietary polysaccharides into monosaccharides, against colonic mucus degraders (*Muciniphila* for example).^[^ ^57^, ^58^ ^]^ These results showed that Rh‐YBS could inhibit pathogenic bacteria and promote probiotic bacteria. The levels of short‐chain fatty acids (SCFA) in the feces of mice from each group were measured by Gas Chromatography‐Mass Spectrometry. The results indicate that DSS reduced SCFA levels compared with the Control group, while Rh‐YBS significantly increased the SCFA levels (*p* < 0.01) (Figure S9, Supporting Information). Notably, the increase in butyric acid in the Rh‐YBS group compared to other groups suggests the proliferation of butyric acid‐producing bacteria,^[^ ^59^, ^60^ ^]^ a well‐known probiotic, which demonstrates the flora‐regulating capability of Rh‐YBS. Overall, Rh‐YBS had the potential to enhance the colonic microenvironment, modulate colonic flora, and contribute positively to the management of UC.

In this study, Rh‐YBS was designed for drug delivery to the submucosa. To confirm these effects, a flora sequencing analysis of colon tissues was conducted. Surprisingly, the analysis revealed that, unlike in feces, the Control group exhibited the lowest flora diversity in the submucosa, while the Model group had the highest diversity in the colon tissue. Treatment with different formulations resulted in a decrease in flora diversity, with the Rh‐YBS group being the most effective (Figure S10a, Supporting Information). Figure S10b (Supporting Information) indicated that the flora diversity of the Control group was markedly different from all other groups, indicating that under normal conditions, there is a lower flora presence in the submucosa. However, after mucosal damage, bacteria can invade the submucosa, highlighting the necessity of delivering drugs to this area. A family‐level square stacking diagram in Figure S10c (Supporting Information) revealed that the probiotic *Lachnospiraceae* decreased,^[^ ^59^, ^60^ ^]^ while pathogenic bacteria such as *Bacteroidaceae* increased following DSS treatment. Rh‐YBS was able to ameliorate this imbalance. Notably, *Lachnospiraceae* includes various butyric acid‐producing bacteria, which supports the conclusions drawn from the previous SCFA study. The species‐level heatmap in Figure S10d (Supporting Information) demonstrated that DSS caused an increase in certain bacteria in the submucosa, which was subsequently reduced by Rh‐YBS treatment. These findings underscore the flora‐regulating capability of Rh‐YBS in colon tissues.

In summary, the therapeutic effect of Rh‐YBS on UC has been validated, and compared to the Lam + Rh‐YBS group, its efficacy appears to be related to the submucosa.

### 2.4 Regulatory Effect of Rh‐YBS via CGRP‐RN

**Figure 4:**
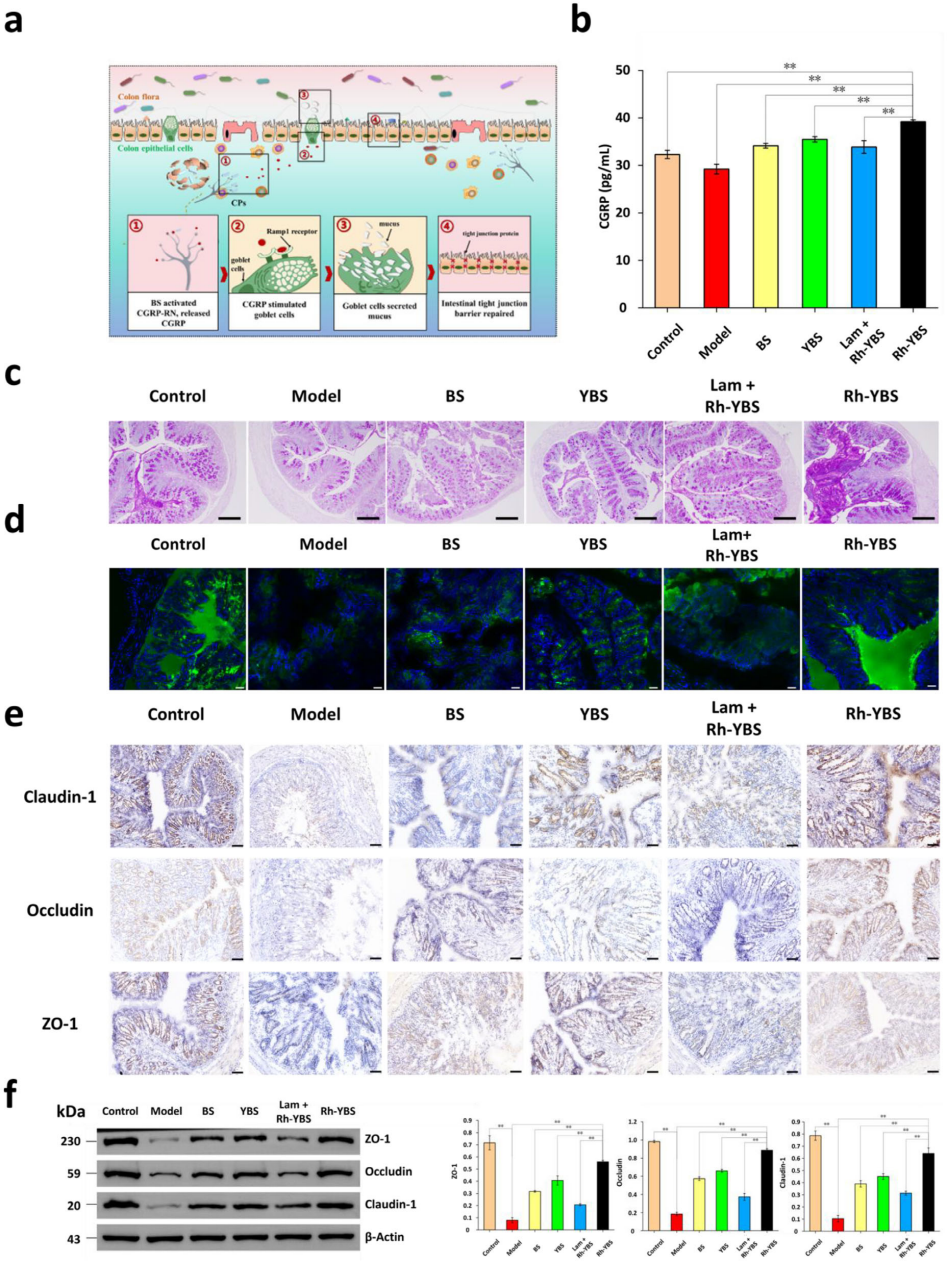
Regulatory effect of Rh‐YBS via CGRP‐RN. a) Schematic illustration of UC treatment procedure via CGRP‐RN. b) Histogram about levels of CGRP content in colons treated among different groups (n = 5). c) PAS stain for mucosa reparation evaluation on colonic sections in different groups. Scale bar: 200 µm. d) Frozen sections of colon tissues. DAPI represented colon tissue, FITC represented mucosa. Scale bar: 50 µm. e) IHC of tight junction protein (Claudin‐1, occluding, ZO‐1) in colons of different groups. Scale bar: 50um. f) Western blot of tight junction protein in colons of different groups (n = 3). Data are mean ± SE. *p < 0.05; **p < 0.01.

In the previous research, we proposed a hypothesis that BS could proliferate within the submucosa, subsequently activating CGRP‐RN to facilitate CGRP. This releasee of CGRP was postulated to stimulate goblet cells, thereby inducing mucus secretion to promote mucosal repair. We hypothesized that this cascade of events would ultimately contribute to the restoration of the colonic tight junction barrier (**Figure** **4**a). This mechanistic hypothesis was investigated through subsequent experimental investigations.

Initial investigations focused on quantifying CGRP expression profiles across experimental groups by ELISA. The colonic tissues from Rh‐YBS‐treated mice exhibited substantially elevated CGRP concentrations compared to control groups (*p* < 0.01; Figure 4b). Of particular significance, this CGRP upregulation was markedly attenuated in the Lam + Rh‐YBS cohort (*p* < 0.01), suggesting the role of M cell‐mediated submucosal delivery in Rh‐YBS bioactivity. These findings suggested Rh‐YBS as an activator of CGRP‐RN signaling pathways.

To elucidate the mucosal repair mechanisms, multifaceted analytical approach was employed. Periodic Acid‐Schiff staining (PAS) revealed dramatic alterations in goblet cell dynamics (Figure 4c). Mice with DSS‐induced colitis displayed severe goblet cells depletion with concomitant mucus hyposecretion. While YBS and Lam + Rh‐YBS treatments partially restored goblet cell populations, the Rh‐YBS administration resulted in both complete goblet cells replenishment and robust mucus production. CLSM was used to characterize the mucosal repair effect of Rh‐YBS. Figure 4d showed that the Rh‐YBS group demonstrated near‐complete restoration of mucosal architecture, closely mirroring healthy control specimens, in contrast to the extensive epithelial damage observed in the Model group. These results supported the role of goblet cells in mucosal repair.

The restoration of mucosal integrity represents a critical therapeutic endpoint, as it prevents pathogenic infiltration and facilitates the reconstruction of the epithelial barrier. To systematically evaluate tight junction restoration, we conducted comprehensive analyses of three pivotal junctional proteins: Claudin‐1, Occludin, and ZO‐1 by immunohistochemistry (IHC). Claudin‐1 primarily regulates the permeability of tight junctions and their selective barrier function, serving as a crucial regulator of paracellular transport. Occludin contributes to the structural stability and dynamic regulation of tight junctions, playing an essential role in barrier integrity and signal transduction. ZO‐1 functions as a scaffold protein that links transmembrane proteins to the cytoskeleton, thereby maintaining both the structural integrity and functionality of tight junctions.^[^ ^61^, ^62^, ^63^, ^64^ ^]^ It was observed that severe disruption of the tight junction barrier occurred in the Model group, with markedly diminished expression of all three proteins. In contrast, Rh‐YBS treatment resulted in near‐complete restoration of junctional architecture, with protein expression patterns closely mirroring those of the Control group (Figure 4e). The expression levels of Claudin‐1, Occludin, and ZO‐1 in the Rh‐YBS group significant increased compared to other treatment groups (*p* < 0.01), approaching physiological levels observed in Control group (*p* > 0.05) by Western blot. These experiments indicated Rh‐YBS‐mediated UC therapy through CGRP‐RN activation. The therapeutic cascade involved included targeted delivery of probiotics to the submucosal layer, localized probiotic proliferation in proximity to CGRP‐RN, and subsequent activation of CGRP‐RN signaling pathways. This mechanistic investigation validates the therapeutic potential of Rh‐YBS and delineates a novel probiotic‐based strategy for UC management through targeted CGRP‐RN modulation.

### 2.5 Contributions of BS to Rh‐YBS

The above investigations had substantiated the therapeutic efficacy of Rh‐YBS in UC treatment through CGRP‐RN activation, however, a critical knowledge gap remained in the mechanistic understanding of this process. As illustrated in Figure S8b (Supporting Information), microbial profiling during UC progression revealed substantial alterations in flora diversity and submucosal bacterial infiltration. However, the existing evidence could not conclusively establish whether CGRP‐RN activation was specifically mediated by BS rather than other bacteria. To address this fundamental question and establish causal relationships, an antibiotic‐treated (ABX) mouse model was employed, which allowed for investigation of BS‐specific effects on CGRP‐RN activation.

The mice were treated as shown in Figure S11a (Supporting Information). Following sacrifice, colonic tissues were aseptically processed, with mucosal layers carefully removed to facilitate precise submucosal sampling. Tissue homogenates were then subjected to BS‐specific plate counting analysis (Figure S11b, Supporting Information). Quantitative analysis revealed significant BS proliferation in the submucosal layer at both 6_th_ and 12_th_ h time points post‐administration, demonstrating successful submucosal colonization by both YBS and Rh‐YBS formulations. Notably, the attenuated BS levels in the Lam + Rh‐YBS group provided direct evidence for M cell‐dependent targeting of Rh‐YBS. Intriguingly, BS populations exhibited a natural decline after 24 h, potentially attributable to the aerobic nature of BS in the hypoxic colonic environment. This temporal pattern suggests minimal residual effects of BS in healthy individuals, highlighting the safety profile of this therapeutic approach. Collectively, these findings demonstrated the successful targeted delivery of Rh‐YBS to the submucosal compartment, while simultaneously addressing potential safety concerns regarding bacterial persistence.

**Figure 5:**
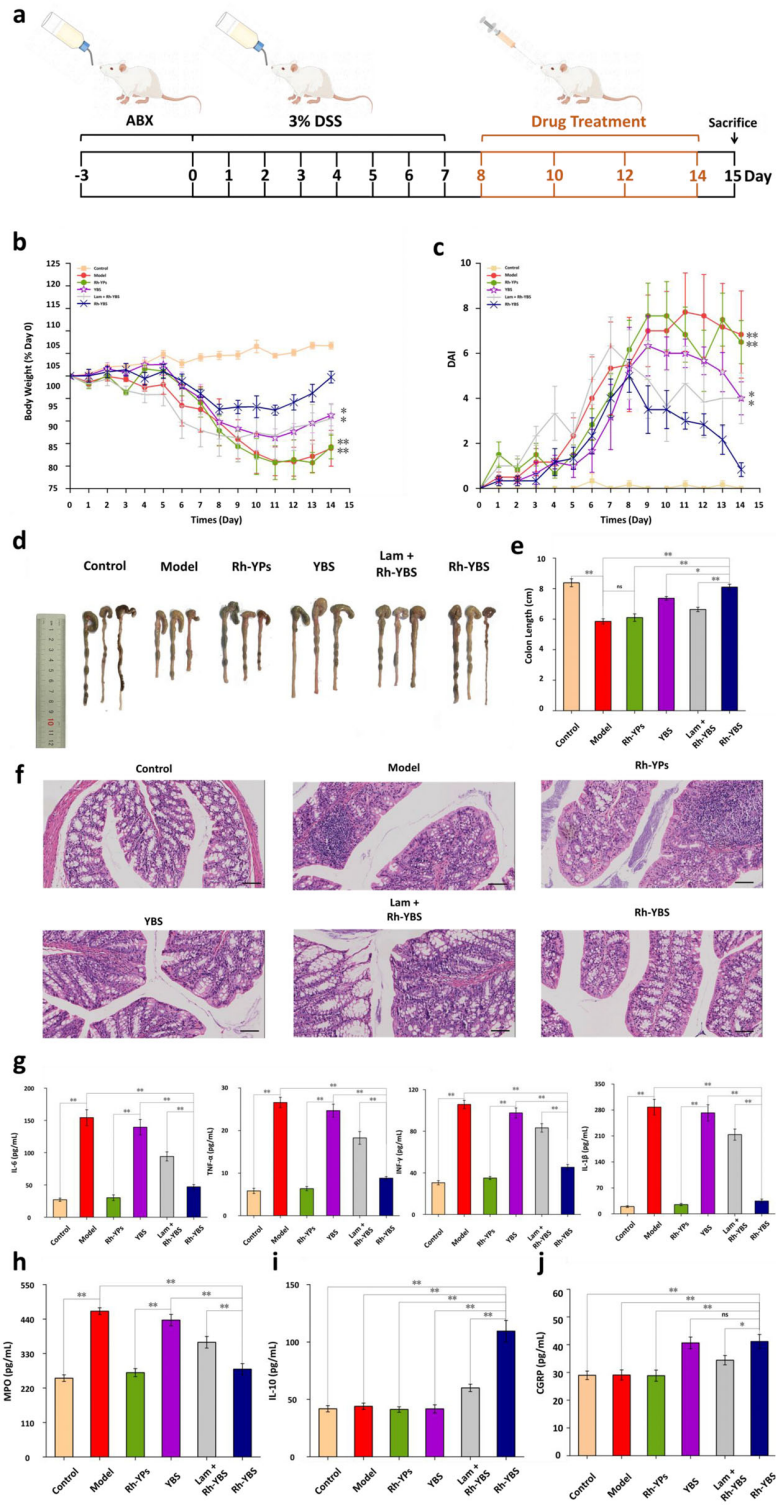
Contributions of BS to Rh‐YBS. a) Therapeutic procedure of different therapeutic formulations to ABX mice. b) Broken‐line graph of body weight varying percent (compared to day 0) with time among different therapeutic formulas (n = 6). c) Broken‐line graph of DAI scores varying with time among different groups (n = 6). d) Images of colon among different groups. e) Histogram of colon length among different group (n = 6). f) H&E staining of colonic sections among different groups. Scale bars: 100 µm. g)–j) Histogram analysis of different groups on the levels of (g) inflammatory cytokines (IL‐6, TNF‐α, IFN‐γ, and IL‐1β), (h) oxidation cytokines (MPO), (i) anti‐inflammatory cytokines (IL‐10), (j) Histogram about levels of CGRP in colons (n = 6). Data are mean ± SE. *p < 0.05; **p < 0.01.

After Rh‐YBS was localized in the submucosa, the contribution of BS to Rh‐YBS in UC treatment was evaluated in the following experiment. Mice were divided into 6 groups and treated with an antibiotic cocktail (1 mg mL^−1^ penicillin, 2 mg mL^−1^ streptomycin, and 0.25 mg mL^−1^ vancomycin) in their drinking water for 3 days to clear the colonic flora. Subsequently, the mice were given DSS water for 7 days. Different preparations were administered via oral gavage according to experimental requirements (**Figure** **5**a). As depicted in Figure 5b, mice in the Model group experienced significant body weight loss of 85.0% (*p* < 0.01). The Rh‐YPs exhibit a disappointing therapy effect, the body weight of mice was 88.0% at 14_th_ day, with no significant diffidence compared to the Model group (*p* > 0.05). Results from the Rh‐YBS group showed that Rh‐YBS restored the body weight of mice to 99.7% at the endpoint, while Lam affected the therapeutic effect of Rh‐YBS (92.0% at endpoint). The Disease Activity Index (DAI) score showed similar trends to the body weight curve, with symptoms of diarrhea and hematuria in mice in the Model group, and symptoms in the Lam + Rh‐YBS group were significantly more severe than in the Rh‐YBS group *(p* < 0.05) (Figure 5c). Colon images and colon length histograms from each group demonstrated that Rh‐YBS could ameliorate UC (Figure 5d,e). H&E image of colon showed that Rh‐YBS group is most effective to ameliorate the symptoms of colonic inflammation (Figure 5f). The tissue images of heart, liver, spleen, lung, and kidney by H&E staining showed no observable damage (Figure S12, Supporting Information). ELISA analysis showed that inflammatory markers (IL‐6, TNF‐α, IFN‐γ, and IL‐1β) and MPO status in the colon (Figure 5g–i) and serum (Figure S13a,b, Supporting Information) were improved by Rh‐YBS, and the level of anti‐inflammatory factors (IL‐10) in the colon (Figure 5h) and serum (Figure S13c, Supporting Information) in the Rh‐YBS group was significantly higher than in other groups (*p* < 0.05). The above data indicated that the Rh‐YPs has no therapy effect to UC compared with Model group (*p* > 0.05), while Rh‐YBS showed a significant difference to Model and Rh‐YPs groups (*p* < 0.01). It was considered that the therapeutic effect of Rh‐YBS on UC is related to BS. Importantly, ELISA results showed that CGRP levels in the colon of the Rh‐YBS group were significantly higher than those in other groups (*p* < 0.01), while Lam led to a decrease in CGRP levels (Figure 5i), indicating that CGRP‐RN was activated by BS. It is worth noted that YBS group also showed a considerable CGRP increase, which may be caused by the proliferation of BS, with no Rh assistance needed in the ABX mice, which were lacking of flora. These findings suggest that the capability of Rh‐YBS to activate CGRP‐RN is associated with BS.

Through comprehensive experimental in this part, two critical aspects of the therapeutic mechanism had been successfully demonstrated: efficient delivery and colonization of BS within the submucosal layer in vivo, and subsequent activation of CGRP‐RN by BS. These findings provide experimental evidence supporting the essential role of BS in mediating the therapeutic effects of Rh‐YBS, thereby establishing solid foundation for its mechanism of action.

### 2.6 Contributions of Rh to Rh‐YBS

While preceding investigations had elucidated the therapeutic efficacy of YPs and Rh‐YBS, and BS functionality, the precise role of Rh in Rh‐YBS remained to be characterized. As demonstrated in section 2.5, Rh appeared to be inactive in the CGRP‐RN pathway in the ABX mouse model, raising questions about its mechanistic involvement. We conducted additional experiments that unexpectedly revealed previously unrecognized functions of Rh.

The antibacterial ability of Rh were tested. We evaluated the inhibitory effects of Rh on pathogenic bacteria and BS using a 96‐well plate assay (Figure S14, Supporting Information). The minimum inhibitory concentration of Rh for BS was approximately 80 µg mL^−1^, and less than 40 µg mL^−1^ for certain common pathogenic bacteria, including *E. coli, Akkermansia muciniphila, Parabacteroides gordonii, and Clostridium perfringens*.^[^ ^65^, ^66^, ^67^ ^]^ These results indicated that Rh was efficient in inhibiting pathogenic bacteria, which helps BS gain a competitive advantage against these common pathogens. To further validate this ability, a plate count of *E. coli* after different treatments was preferred. As shown in Figure S15 (Supporting Information), the colony numbers of *E. coli* in the Rh group and the Rh + BS group were significantly reduced (*p* < 0.05) after 6 h of incubation, while only the Rh + BS group shown significant inhibition of *E. coli* after 12 h (*p* < 0.05). These results demonstrated that Rh inhibits *E. coli* at 6_th_ h, helping BS compete effectively with *E. coli* during subsequent incubation, suggesting that the function of Rh is to regulate the balance between probiotic and pathogenic bacteria. However, the short‐term effect of Rh in the experiment raises the question of whether Rh needs to be administered continuously. To address this, further experiments were conducted.

**Figure 6:**
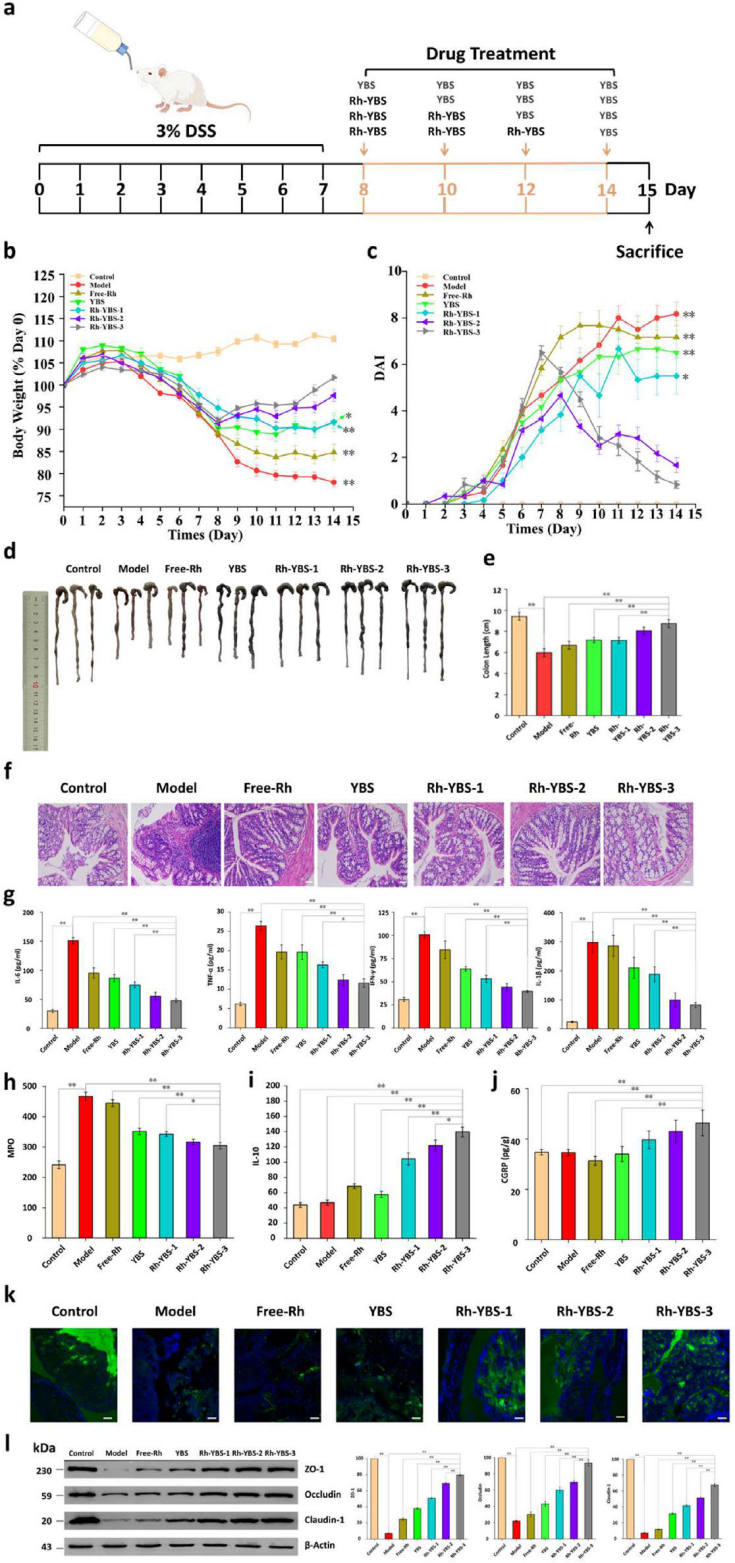
Contributions of Rh to Rh‐YBS. a) Therapeutic procedure of different therapeutic formulations to DSS mice. b) Broken‐line graph of body weight varying percent (compared to day 0) with time among different therapeutic formulas (n = 6). c) Broken‐line graph of DAI scores varying with time among different groups (n = 6). d) Images of colon among different groups. e) Histogram of colon length among different groups (n = 6). f) H&E staining of colonic sections among different groups. Scale bars: 100 µm. g)–j) Histogram analysis of different groups on the levels of (g) inflammatory cytokines (IL‐6, TNF‐α, IFN‐γ, and IL‐1β), (h) oxidation cytokines (MPO), (i) anti‐inflammatory cytokines (IL‐10), (j) Histogram about levels of CGRP in the colon (n = 6). k) Frozen sections of colon tissue. DAPI represented colon tissue, FITC represented mucosa. Scale bar: 50 µm. l) Western blot of tight junction protein in colon of different groups (n = 3). Data are mean ± SE. *p < 0.05; **p < 0.01.

Mice were divided into 7 different groups and treated as shown in **Figure** **6**a. The body weight (Figure 6b) and DAI (Figure 6c) of the mice exhibited similar trends to the previous in vivo experiment. The health of the mice improved around the 10_th_ day, with the Rh‐YBS‐3 group showing a significantly better therapeutic effect compared to other groups (*p* < 0.05). The colon length of the mice in each group indicated that the colon in the Rh‐YBS‐3 group was similar to that of the Control group (*p* = 0.18) (Figure 6e). H&E staining results demonstrated that groups treated with Rh‐YBS had improved colon inflammation (Figure 6f). The ELISA test results showed that the levels of most inflammatory factors (IL‐6, TNF‐α, IFN‐γ, IL‐1β, and MPO) in the colon (Figure 6g–i) and serum (Figure S16a,b, Supporting Information) were significantly lower in the Rh‐YBS‐3 group compared to other DSS‐treated groups, except the Rh‐YBS‐2 group (*p* < 0.05), while the IL‐10 level in the colon (Figure 6j) and serum (Figure S16c, Supporting Information) of the Rh‐YBS‐3 group was significantly higher than that in other groups (*p* < 0.05). The ELISA results for CGRP showed a slightly different pattern: the CGRP level in the Rh‐YBS‐3 group was the highest, with a significant difference compared to all other groups (*p* < 0.01), except the Rh‐YBS‐1 and Rh‐YBS‐2 groups (Figure 6j). This phenomenon suggests that without the assistance of Rh, BS could not establish itself in the submucosa sufficiently to activate CGRP‐RN. The results of colon fluorescence staining supported this hypothesis, as shown in Figure 6k; all three groups treated with Rh‐YBS had intact mucosa. Lastly, the results for tight junction proteins (Claudin‐1, Occludin, and ZO‐1) showed that the Rh‐YBS‐3 group had the highest levels, significantly different from other groups (Figure 5l). Figure S17 (Supporting Information) confirmed again that these treatments caused no obvious damage to different organs. These results revealed that the Rh‐YBS‐3 treatment regimen consistently outperformed both Rh‐YBS‐2 and Rh‐YBS‐1 groups across multiple therapeutic indices. This dose‐dependent enhancement in therapeutic efficacy suggested the contributions of Rh to Rh‐YBS.

### 2.7 The Preventive Efficacy of Rh‐YBS in UC

Recognizing the chronic and recurrent nature of UC, where patients in remission maintain substantial risk of disease recurrence,^[^ ^59^ ^]^ a specific experiment was designed to evaluate the prophylactic efficacy of Rh‐YBS. This investigation aimed to assess the therapeutic potential of Rh‐YBS in maintaining remission and preventing disease relapse, addressing a critical unmet need in UC management.

**Figure 7:**
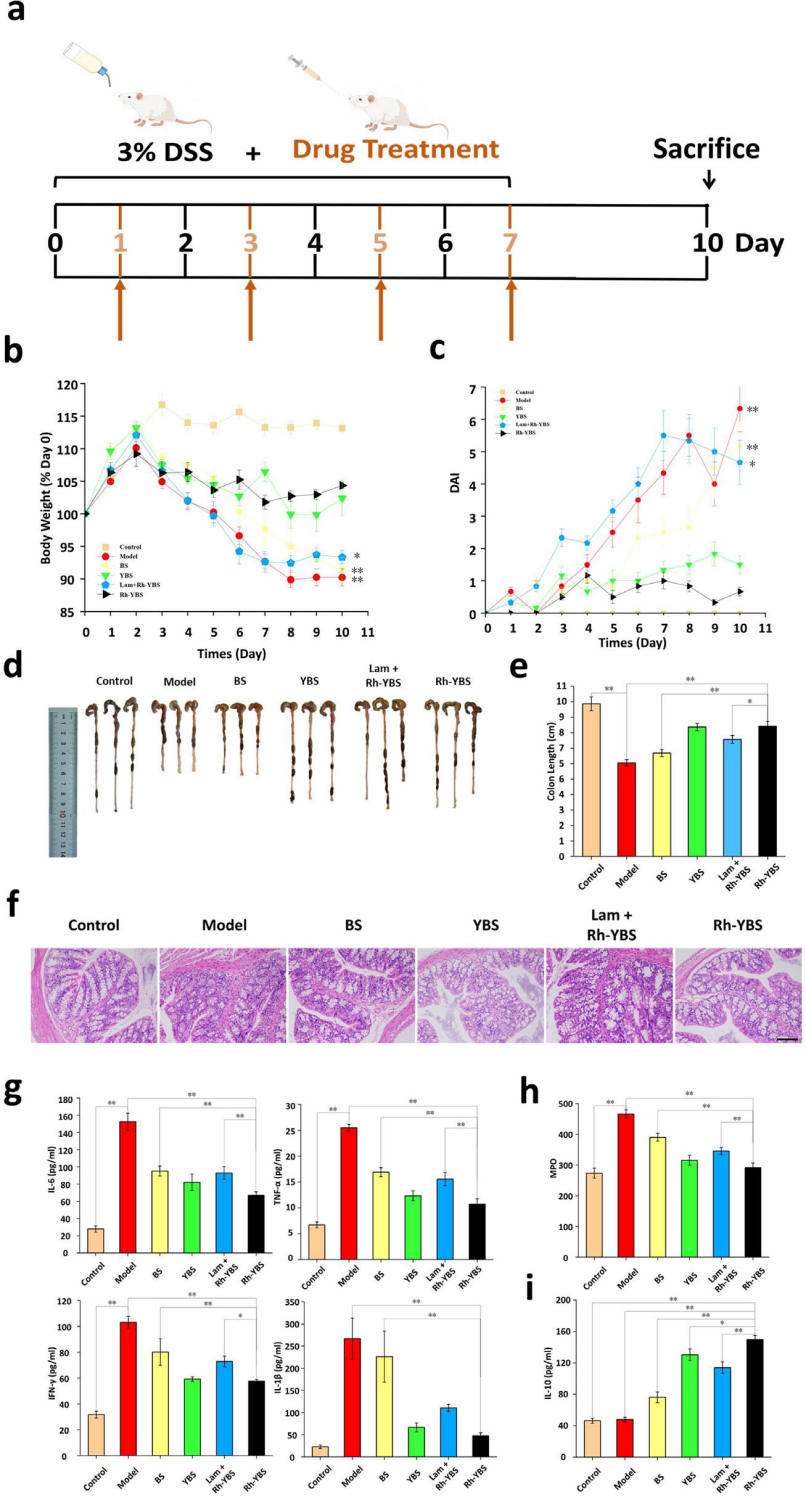
Evaluation of the Preventive efficacy of Rh‐YBS in UC. a) Therapeutic procedure of different therapeutic formulations to mice with high‐risk of UC. b) Broken‐line graph of body weight varying percent (compared to day 0) with time among different therapeutic formulas (n = 6). c) Broken‐line graph of DAI scores varying with time among different groups (n = 6). d) Images of colons among different groups. e) Histogram of colon length among different groups (n = 6). f) H&E staining of colonic sections among different groups. Scale bars: 100 µm. g)–i) Histogram analysis of different groups on the levels of (g) inflammatory cytokines (IL‐6, TNF‐α, IFN‐γ, and IL‐1β), (h) oxidation cytokines (MPO), (i) anti‐inflammatory cytokines (IL‐10) in the colon (n = 6). Data are mean ± SE. *p < 0.05; **p < 0.01.

The experimental design involved six groups of mice receiving 3% DSS every two days, with treatments administered orally every other day until sacrificed on the 10_th_ day post‐modeling (**Figure** **7**a). Notably, the YBS, Lam + Rh‐YBS, and Rh‐YBS groups exhibited comparable therapeutic efficacy, all demonstrating significant improvements compared to the Model group (*p* < 0.01), Longitudinal monitoring revealed that DSS‐induced body weight loss commenced at 3_rd_ day, with treatment groups showing rapid stabilization of body weight curves, ultimately maintaining significantly higher weights than the Model group at the endpoint (*p* < 0.05; Figure 7b). The DAI scores confirmed consistently lower inflammation levels in all treatment groups compared to both Model and BS groups (Figure 7c). Morphometric analysis of colon length revealed similar measurements (≈8.2 cm) across YBS, Lam + Rh‐YBS, and Rh‐YBS groups (*p* > 0.05), significantly exceeding the Model group's average of 6.0 cm (*p* < 0.01; Figure 7d,e). Histopathological evaluation through H&E staining demonstrated substantial improvement in colonic tissue architecture across all treatment groups contained YBS compared to the Model group (Figure 7f), with no evidence of treatment‐related organ toxicity (Figure S18, Supporting Information). Biochemical analyses revealed reduced MPO activity and proinflammatory cytokine levels, coupled with elevated anti‐inflammatory factors in both YBS, Lam + Rh‐YBS, and Rh‐YBS groups (Figure 7g–i), which was in consistent with the cytokine profiles (Figure S19, Supporting Information).

The comparable preventive efficacy across YBS, Lam + Rh‐YBS, and Rh‐YBS groups may be attributed to intact mucosal barriers limiting Rh‐YBS penetration and subsequent CGRP‐RN activation in healthy tissues. Previous studies have documented BS's multifaceted protective mechanisms, including pathogen inhibition.^[^ ^68^ ^]^ and promotion of intestinal cell differentiation,^[^ ^19^, ^69^ ^]^ which may contribute to UC prevention. These findings collectively suggest that while Rh‐YBS demonstrates preventive potential, its mechanism of action in prophylactic settings may differ from therapeutic contexts, warranting further investigation into mucosal barrier‐dependent drug delivery dynamics.

## 3 Conclusion

This study presents the development and validation of an innovative oral drug delivery system targeting the colonic submucosa for UC treatment. The Rh‐YBS complex represents a sophisticated therapeutic platform where each component executes distinct yet complementary functions: BS serves as the bioactive core, demonstrating CGRP‐RN activation, microbiota modulation, and cellular differentiation capabilities; YPs function as the precision delivery vehicle, providing gastric protection, M cells targeting, and facilitating submucosal BS proliferation; while Rh acts as an antimicrobial adjuvant, enhancing BS's competitive advantage against pathogenic flora. This multifaceted approach resulted in comprehensive therapeutic benefits, including improved DAI scores, colon morphology, mucosal integrity, tight junction restoration, and microbiota homeostasis in the UC model.

While our findings establish the feasibility of submucosal‐targeted drug delivery for UC management, several critical questions warrant further investigation. The clinical translatability of this delivery system requires rigorous evaluation, particularly regarding scalability and manufacturing considerations. The immunomodulatory properties of Rh and its potential influence on BS proliferation dynamics in the submucosal microenvironment represent another important research direction. It is worth noting that the comparable preventive efficacy observed between Rh‐YBS and YBS formulations suggests context‐dependent roles for Rh, potentially limiting its necessity in prophylactic applications. These unresolved aspects provide avenues for future research to optimize this therapeutic platform for clinical application.

## Conflict of Interest

The authors declare no conflict of interest.

## Author Contributions

L. Li., L. Dai., and M. Lin contributed equally to this work. L. Li., F. Gao., and D. Lin developed the overall methodology and conducted the theoretical analysis of this study. L. Lin., L. Dai., and M. Lin performed the majority of the experiments and data analysis. S. He., H. Du., Y. Wang., F. Zhang., Q. Wang., L. He., and K. Wu carried out bacterial culture tasks. S. Tao., X. Sun., X. Huang., and H. Liu provided assistance in the animal studies. J. You., M. Zhang., C. Fu., H. Tu., N. Ye., and J. Liu contributed to the manuscript preparation by providing editorial support.

## Supporting information



Supporting Information

## Data Availability

Research data are not shared.
