# Supplementary material for: Colonic Submucosa Targeted Delivery of Probiotic and Rhein for Ulcerative Colitis Treatment
Source: Adv Sci (Weinh). 2025 May 9;12(30):2409711. doi: 10.1002/advs.202409711 (PMC12376669; doi:10.1002/advs.202409711)
Supplement: Supplementary file 1 — Supporting Information [file ADVS-12-2409711-s001.docx]

**Colonic Submucosa Targeted Delivery of Probiotic and Rhein for Ulcerative Colitis Treatment**

*Lingqiang Li ^#^, Linxin Dai^#^, Meisi Lin^#^, Shuang He, Hongye Du, Dasheng Lin^*^, Yanbin Wang, Fenglian Zhang, Sian Tao, Xiaoluo Sun, Xinggui Huang, Haihui Liu, Qian Wang, Lingling He, Kunhe Wu, Jieshu You, Minyue Zhang, Chaomei Fu, He Tu, Naijing Ye^*^, Jibin Liu^*^, Fei Gao ^*^*

**Experimental section**

*Materials, strains, and animals*

Brewer's yeast was obtained from Angel Co. (Hubei, China). Rh and dimethyl sulfoxide (DMSO) were sourced from J&K Scientific Co. (Beijing, China). cyanine 5 bisacid-N-hydroxy succinimide (Cy5-NHS), anti-ulex europaeus lectin 1-fluorescein isothiocyanate (UEA-1-FITC), Calcofluor-White, and DAPI (4',6-diamidino-2-phenylindole) were acquired from MeilunBio Co., Ltd. (Dalian, China). Sodium dextran sulfate (DSS) was purchased from Aladdin Reagent Co. (Shanghai, China). Mouse ELISA kits for Interleukin (IL)-1β, IL-6, IL-10, tumor necrosis factor-α (TNF-α), Interferon (IFN-γ), Myeloperoxidase (MPO), and calcitonin gene-related peptide (CGRP) were obtained from Sizhengbai Biotechnology Co., Ltd. (Beijing, China). All chemicals used were used as received without further purification.

*Bacillus subtilis* ATCC6633, *Akkermansia muciniphila* BNCC341917, *Escherichia coli* BNCC336902, *Parabacteroides gordonii* BNCC366356, and *Clostridium perfringens* BNCC317723 were stored at -80℃ in our laboratory.

Male ICR mice (approximately 30 g, 6-8 weeks old) and related supplies were acquired from SPF Biotechnology Co., Ltd. (Beijing, China; approval number: SCXK(Jing)2019-0010), and were housed in specific pathogen-free conditions, with six mice per cage, provided deionized water daily, and maintained at a temperature of 25 ± 1°C. All mice underwent a two-day acclimatization period prior to the experiment. The animal research protocol was approved by the Animal Ethics Committee of Chengdu University of Traditional Chinese Medicine (2024011).

*Preparation of YPs*

To prepare YPs, 100 g of brewer's yeast was suspended in 1000 mL of deionized water containing 1 M sodium hydroxide. The mixture was heated to 80°C for 1 h and then centrifuged at 3000 rpm for 10 min. The pellet was washed twice with deionized water, resuspended in hydrochloric acid (pH = 4), and incubated at 60°C for 1.5 h. After incubation, the sample was centrifuged at 3000 rpm for 10 min and washed thoroughly twice with deionized water. The pellet was then washed four times with isopropyl alcohol and twice with acetone. After final centrifugation at 3000 rpm for 10 min, the precipitate was freeze-dried following a 12-h pretreatment at -20℃ to yield YPs. ^[1, 2]^

*Preparation of Rh-YPs*

Rh-YPs were obtained by electrostatic force-driven self-deposition and solvent hydration/lyophilization method. Initially, 15 mg of Rh was dissolved in 5 mL of DMSO. To this solution, 300 mg of YPs were added, followed by the addition of 15 mL of deionized water. The mixture was stirred at room temperature for 24 h. The precipitate was collected by centrifugation at 7000 rpm for 5 min. Then, the mixture was transferred to a 1000 Da dialysis bag (Millipore, USA) and dialyzed against deionized water, with the water changed every 4 h, for 2-3 days. Finally, precipitate was lyophilized to produce Rh-YPs. ^[1, 2]^

*Characterization of Rh-YPs*

HPLC (LC-45202-46, Shimadzu, Japan), equipped with a C18 column, was utilized to analyze the content of Rh. Methanol/0.1% phosphoric acid (85/15, *v/v*) was selected as the mobile phase. The flow rate was maintained at 1 mL/min, and the detection wavelength was set at 254 nm. ^[3]^ A standard solution of Rh was prepared by dissolving the reference substance in DMSO/ phosphate-buffered saline (PBS) (1:19, *v/v*) to achieve a concentration of 384 μg/mL. This standard solution was further diluted to concentrations of 192 μg/mL, 96 μg/mL, 48 μg/mL, 24 μg/mL, 12 μg/mL, 6 μg/ mL, and 3 μg/mL. The supernatant in Rh-YPs incubation was collected, filtered through a 0.22 μm microporous membrane, and used to prepare the Rh sample solution. The encapsulation rate of Rh on YPs was calculated using the formula:

Encapsulation rate = [(Rh total) -(Rh sample)]/ (Rh total) ×100%

The ultraviolet spectrum (UV) was also used to characterize Rh-YPs. Approximately 20 mg of Rh-YPs was spread and flattened on the surface of barium sulfate and analyzed using an ultraviolet spectrophotometer (UH4150, Japan). The measurement range was 200-800 nm with a wavelength accuracy of 1 nm.

An infrared spectrometer (IR) was used to further characterize Rh-YPs. Approximately 1–2 mg of Rh-YPs was mixed with 200 mg of pure KBr. The mixture was placed in a mold and pressed into transparent sheets using an oil press. These sheets were analyzed using a Fourier transform infrared spectrometer (Thermo Scientific Nicolet 6700), with a wave number range of 4000 to 400 cm^-1^, 32 scanning times, and a resolution of 4 cm^-1^. ^[4]^

*Culture of bacteria*

BS strains and *E. coli* strains were inoculated onto Luria-Bertani (LB) solid medium and incubated at 37℃ overnight. A single correct colony was selected and inoculated into LB liquid medium, which was then incubated at 37°C overnight with shaking at 150 rpm. Subsequently, 10 μL of the culture solution was transferred into 10 mL of LB liquid medium and incubated at 37℃ for 6 h. ^[5]^

*Akkermansia muciniphila* strains were inoculated onto BHL solid medium and incubated at 37°C for 72 h under low oxygen conditions (10% CO_2_, 5% O_2_, 85% N_2_). A single correct colony was selected and inoculated into BHL liquid medium, then incubated at 37°C for 48 h under low oxygen conditions. Following this, 10 μL of the culture solution was transferred into 10 mL of BHL liquid medium and incubated at 37°C for another 48 h under low oxygen conditions.

*Parabacteroides gordonii* strains were inoculated onto TSB solid medium supplemented with 5% sheep blood and incubated at 37℃ for 48 h under low oxygen conditions. A single correct colony was selected and inoculated into TSB liquid medium supplemented with 5% sheep blood, then incubated at 37℃ for 36 h under low oxygen conditions. Afterward, 10 μL of the culture solution was transferred into 10 mL of TSB liquid medium supplemented with 5% sheep blood, and incubated at 37°C for 36 h under low oxygen conditions.

*Clostridium perfringens* strains were inoculated onto FT solid medium and incubated at 37℃ overnight under low oxygen conditions. A single correct colony was selected and inoculated into FT liquid medium, then incubated at 37℃ overnight under low oxygen conditions. Finally, 10 μL of the culture solution was transferred into 10 mL of FT liquid medium and incubated at 37°C for 10 h under low oxygen conditions.

*Preparation of Rh-YBS*

Rh-YBS was produced using the extrusion method. 2 mg of Rh-YPs were suspended in 1 mL of cold PBS and treated with ultrasonic waves for 15 min. It was then mixed with BS culture solution (2 × 10^9^ CFUs) and incubated at 37°C for 1 h with shaking at 150 rpm. The mixture was then transferred to a mini extruder (Nexstar Nano Technology, Shanghai) and extruded 20 times through a polycarbonate porous membrane with a pore size of 5 μm, then product was centrifuged at 7000rpm for 5min, the precipitated Rh-YBS was obtained. ^[6]^

*Characterization of Rh-YBS*

To observe the external features of Rh-YBS, a Scanning Electron Microscope (SEM) was used. Rh-YBS was treated with glutaraldehyde fixative (Solarbio Science & Technology Co., Ltd., Beijing) for 12 h, placed on conductive adhesives, and coated with metal using a sputter coater (Oxford Quorum SC7620, England) for 45 S. The sample was then observed and imaged under an SEM (ZEISS GeminiSEM 300, Germany).

For further observation of the external features of Rh-YBS, a TEM was employed. Rh-YBS was treated with glutaraldehyde for 12 h, fixed with osmium tetroxide for 2 h, and rinsed with Phosphate Buffer (PB, pH = 7.4) for 15 min, repeated three times. The sample was then subjected to a gradient dehydration process using acetone at different concentrations, embedded in paraffin at 37°C overnight, and polymerized at 60°C for 48 h. The embedded sample was then sectioned to 70 nm, stained with uranyl acetate for 10 min and lead citrate for 8 min, and observed and imaged under a TEM (Hitachi HT7800, Japan) after drying.

To obtain a comprehensive view of Rh-YBS, a Confocal Laser Scanning Microscope (CLSM) was utilized. BS was stained with Cy5-NHS, and Rh-YPs were stained with calcofluor white. The samples were observed and imaged using a CLSM (TCS SP8 SR, Leica, Weztlar, Germany). The excitation wavelengths were set at 350 nm and 650 nm; the emission wavelengths were set at 400-450 nm and 600-700 nm.

*Verification of Rh-YBS's stability in stomach*

The stability of Rh-YBS in the stomach was evaluated using simulated gastric fluid (SGF). BS, BS + Rh, YBS, and Rh-YBS contain 10^8^ CFUs BS was added in SGF (pH = 1.2), at 0 h, 0.5 h, 1h and 2h after incubation, 100 μL samples in every group were decanted, and the samples were resuspended with 900 μL of LB liquid medium. This was followed by a gradient dilution in a 1:10 ratio. From these dilutions, 100 μL of each solution was plated on LB solid medium and incubated at 37℃ for 12 h. Plates with colony counts ranging from 20 to 300 were selected for enumeration. As a control, BS (10^8^ CFUs) underwent the same procedure as Rh-YBS.

*Verification of Rh-YBS's targeting and retention in colon*

The targeting and retention capabilities of Rh-YBS in the colon were assessed using an In Vivo Imaging System (IVIS). Mice were randomly divided into five groups. One group served as the control, while the others received 3% DSS in their drinking water for 7 days. Subsequently, mice in the Control group were orally administered by 0.2 mL of Rh-YBS, mice in the other groups received 0.2 mL of BS, 0.2 mL YBS, 0.2 mL of 0.1% Lam + 0.2 mL of Rh-YBS, and 0.2 mL of Rh-YBS. All BS preparations were standardized to 1.0 × 10^8^ CFUs and labeled with Cy5-NHS. The mice were sacrificed at 3_rd_, 6_th_, 9_th_, and 12_th_ h post-administration, and their digestive tracts, excluding the stomach, were harvested for imaging with the IVIS (Series III 900/1700-L, China). In subsequent tissue-level studies, two groups of mice were established: one received deionized water, and the other received deionized water supplemented with 3% DSS for 7 days. Their colons were harvested, opened longitudinally, and sectioned into 2-3 cm segments. Colons from the DSS-untreated group were immersed in Rh-YBS solution, whereas those from the DSS-treated group were immersed in preparations of BS, Lam + Rh-YBS, and Rh-YBS at 37℃ for 4 h. The colons were then rinsed 2-3 times with PBS, observed, and imaged using IVIS

*Bacterial translocation by Microfold cells in vitro*

Microfold cells *in vitro* were cultured to assess the penetrative ability of Rh-YBS. Caco-2 cells were maintained at 37°C under a 5% CO_2_ atmosphere in DMEM enriched with 20% FBS and 1 % antibiotic/antimycotic solution. Raji cells were cultured in RPMI 1640 medium supplemented with the same concentrations of FBS and antibiotic/antimycotic solution. Volumes of 0.1 mL and 1.5 mL of DMEM supplemented with 10% FBS were added to the apical and basolateral chambers of the trans-well inserts, respectively. The inserts were preincubated for 30 min in a CO_2_ incubator. Subsequently, Caco-2 cells (5 × 10^5^) were seeded on the apical side of the trans-well inserts (1.12 cm^2^; pore size, 3 μm) and cultured for 14 days, with medium changes every 2 days. After 14 days, Raji cells (5 × 10^5^ cells) in a RPMI 1640/Dulbecco’s modified Eagle’s medium (1: 2) mixture were added to the basolateral chamber and cultured for an additional 7 days. Then, 0.2 mL of BS, 0.2 mL of Lam + 0.2 mL of Rh-YBS, and 0.2 mL of Rh-YBS were introduced into the apical side of the transwell inserts, respectively. The plates were incubated at 37°C for 4 h, after which the medium from the basolateral chamber was collected, subjected to gradient dilution, plated on Bacillus Cereus Selective (BCS) solid medium, incubated at 37℃ for 12 h, the colony numbers were counted. ^[6]^

*Verification of proliferation promoting ability of YPs on BS*

YPs, a natural large-molecular polysaccharide, can be degraded by β-glucanase, producing substrates utilizable by BS. To test this, various media were prepared: 10 mL of NB liquid medium, 10 mL of NB liquid medium + 1% β-glucanase, 10 mL of NB liquid medium + 2 mg of YPs, and 10 mL of NB liquid medium + 2 mg of YPs + 1% β-glucanase. A 100 μL aliquot of the BS culture was added to each medium, incubated at 37℃ with shaking at 150 rpm for 6 h. Subsequently, the media were subjected to gradient dilution, plated onto LB solid medium, incubated at 37℃ for 12 h, and the colony numbers were counted. ^[5]^

*Evaluation of treatment efficacy of Rh-YBS for UC in vivo*

Mice were randomly divided into five groups and exposed to 3% DSS in drinking water for 7 days, followed by oral administration of 0.2 mL PBS (Model group), 0.2 mL BS (BS group), 0.2 mL YBS (YBS group), 0.2 mL 0.1% Lam + 0.2 mL Rh-YBS (Lam + Rh-YBS group), and 0.2 mL Rh-YBS (Rh-YBS group) every two days. The BS concentration for all groups was 10^8^ CFUs. Additionally, a control group without DSS treatment received 0.2 mL PBS every two days. Mice were sacrificed on the 15_th_ day post-DSS treatment. During this period, records the body weight changes (0–4), fecal consistency (0–4), and fecal bleeding levels (0–4) of mice. Upon sacrifice, the colonic lengths were measured. Parts of the colons and primary organs (heart, liver, spleen, lungs, and kidneys) were harvested, fixed in 4% paraformaldehyde, embedded in paraffin, and sectioned into 5 μm slices for H&E and PAS staining. Other colon samples, along with serum and feces, were collected for further experimentation. ^[7]^

*Enzyme-linked immunosorbent assay*

Levels of cytokines, MPO, and CGRP in the colon or serum were quantified using ELISA. One milligram of colon tissue was weighed and homogenized in a 1:9 (*m/v*) ratio with homogenization medium (0.9% physiological saline recommended), under ice water bath conditions. Afterward, the homogenates were centrifuged at 2500-3000 rpm for 10 min. The supernatant was then used to detect levels of IL-6, TNF-α, INF-γ, IL-1β, MPO, IL-10, and CGRP using respective ELISA kits. Levels of IL-6, TNF-α, IFN-γ, IL-1β, MPO, and IL-10 in the serum were also quantified. ^[8]^

*Fluorescent staining*

To assess the mucosal status, colons were sectioned into 1 cm segments, embedded in OCT (optimal cutting temperature compound), and frozen at -20℃. These were then sectioned into 20 μm slices. Once dried, the slices were stained with DAPI for 15 min and UEA-1-FITC for 15 min. The stained slices were observed and imaged under a CLSM, with the excitation wavelengths set at 350 nm and 488 nm, and emission wavelengths set at 400-450 nm and 500-550 nm, respectively. ^[9]^

*Immunohistochemistry staining*

To evaluate the expression of ZO-1, Occludin, and Claudin-1. Immunohistochemistry (IHC) was employed. The colon sections were routinely dewaxed in deionized water, incubated with 3% hydrogen peroxide solution for 10–15 min at room temperature, and then rinsed with deionized water for 10 min. The sections were blocked in PBS for 10 min. This was followed by the addition of primary antibody solution dropwise, and incubation for 1–2 h at 37°C. A biotin-labeled secondary antibody solution was then applied dropwise and incubated at 37°C for 10-20 min. Subsequently, horseradish peroxidase or alkaline phosphatase-labeled streptavidin solution was added dropwise and incubated for 10–30 min at 37°C. The sections were rinsed three times with PBS for 5 min each. Color development was conducted for 3–15 min. Images of the tissue were captured by a microscope.

*Western blot*

Total protein was extracted from colon tissue using RIPA lysis buffer (BL504A, biosharp, China) with 100mM PMSF (BL507A, biosharp, China) and protein phosphatase inhibitor (G2007-1ML, Servicebio, China). The total protein concentration in the lysed sample was quantified with a BCA assay kit (BL521A, biosharp, China). Proteins were boiled in 5×SDS-PAGE loading buffer (BL502B, biosharp, China) and separated by 12% SDS-PAGE. After SDS - PAGE, the proteins were transferred to PVDF membranes (IPVH00010, Merck Millipore, USA) and then blocked in 5% milk for 1 h. Rabbit polyclonal antibody anti-PI3K p110a (110 kDa, 4249, CST, China) was used at 1:1000. Rabbit anti-AKT antibodies (56 kDa, ab179463, Abcamm, China) were used at 1:5000 dilution. Rabbit anti-β-actin antibodies (43 KDa, AF7018, Affnity, China) were used at 1:5000 dilution. Western blot quantification was performed using the Image J software. All western blot analyses were run with biological triplicates (n = 3). ^[10]^

*16S rRNA sequencing and SCFAs analysis*

Total genomic DNA was extracted from feces and submucosal samples using the CTAB method. Samples were transferred to an aseptic tube immediately post-sacrifice and stored at -80℃. DNA concentration and purity were assessed on 1% agarose gels. Based on the concentration, DNA was diluted to 1 ng/µL using sterile water. Following extraction, the V3-V4 variable regions of the 16S rRNA gene were amplified using primers 341F (5’-CCTAYGGGRBGCASCAG-3’) and 806R (5’-GACTACNNGGGTATCTAAT-3’) via PCR. The reactions utilized 15 µL of Phusion® High-Fidelity PCR Master Mix (New England Biolabs). Sequencing libraries were constructed using the NEB Next® Ultra DNA Library Prep Kit (Illumina, USA). Library quality was evaluated on an Agilent 5400 (Agilent Technologies Co Ltd., USA), sequenced on an Illumina NovaSeq platform, producing 250 bp paired end reads. ^[11]^

For fecal SCFAs content analysis, 200 mg of feces was homogenized in 1% HCl for 1 min. The homogenate was centrifuged at 3000 rpm for 1 min, and the supernatant was acidified using HCl (pH = 0). Each sample underwent extraction at 4℃ with an equal volume of diethyl ether. The samples were then treated with 1-tertbutyl-dimethylsilyl-imidazole at 60℃ for 30 min and analyzed by Gas Chromatography-Mass Spectrometry (GC-MS, Agilent/5975C, USA).

*Evaluation of the contribution of BS in vivo*

To demonstrate that BS can be delivered to the submucosa, antibiotic (ABX)-treated mice were utilized. Mice were randomly divided into six groups and water was withheld from 1:00 pm to 7:00 pm. They were then administered antibiotics (1 mg/mL of penicillin, 2 mg/mL of streptomycin, and 0.25 mg/mL of vancomycin). After the antibiotics were exhausted, deionized water was provided; this cycle was repeated three times. Subsequently, one groups of ABX-treated mice were chosen as Control, the other five groups were DSS treated and them orally administered 0.2 mL of PBS, 0.2 mL of Rh-YPs, 0.2 mL YBS, 0.2 mL of 0.1% Lam + 0.2 mL Rh-YBS, and 0.2 mL Rh-YBS at 6_th_, 12_th_, and 24_th_ h post-administration. The colons were opened longitudinally and the mucosa removed in a biological safety cabinet (HFsafe-1200LC A2, China). Colon tissue (1 mg) was then homogenized in 1 mL sterile PBS for 2 min. The homogenized samples were gradient diluted, plated on BCS solid medium, and the colony number of BS counted.

To further verify the contribution of BS in Rh-YBS, ABX-treated mice were divided into six groups. The control group was given deionized water, while the other three groups received 3% DSS in deionized water for seven days. Subsequently, the Control group was orally administered PBS every two days, and the other groups were administered 0.2 mL PBS (Model group), 0.2 mL Rh-YPs (Rh-YPs group), 0.2 mL YBS (YBS group), 0.2 mL 0.1% Lam + 0.2 mL Rh-YBS (Lam + Rh-YBS group), and 0.2 mL Rh-YBS (Rh-YBS group) every two days. The health statuses of the mice were monitored and they were sacrificed on the 15_th_ day. Colons, serum, feces, and primary organs (heart, liver, spleen, lungs, and kidneys) were collected for further experiments.

*Evaluation of the contribution of Rh in vitro*

The antibacterial activity of Rh in vitro was determined using the microbroth dilution method. A total of 12.8 mg of Rh was dissolved in 1 mL DMSO via ultrasonication and subjected to a gradient dilution at a ratio of 1:2. Then, 10 μL of the Rh solution was added to each well of a 96-well plate, with three replicates per concentration. A mixture of 190 μL of BS (1.0 × 10^5^ CFUs) in LB liquid medium was added to the Rh solution. Additionally, 200 μL of LB medium was added for the negative control group, and 200 μL of LB liquid medium containing BS (5 × 10^5^ CFUs) was added for the positive control group. The plate was incubated at 37°C for 12 h to determine the MIC of Rh against BS. The MIC of Rh against other bacteria was tested using a similar method.

To evaluate the flora regulation ability of Rh *in vitro*, the following study was conducted: E. coli was incubated in LB medium with and without 400 μg of Rh, BS, and a combination of 400 μg Rh + BS at 37℃. After 6 and 12 h, the cultures were gradient diluted and the colony numbers counted. This method was also applied to BS alone. By assessing changes in the colony numbers of *E. coli* and BS, the flora regulation ability of Rh was analyzed.

*Evaluation of the contribution of Rh in vivo*

Mice were randomly divided into six groups and administered deionized water with 3% DSS for 7 days. Subsequently, the Model group was orally administered PBS on the 8_th_, 10_th_, 12_th_, and 14_th_ days; the Free-Rh group received Rh on the same days; the YBS group received YBS; the Rh-YBS-1 group was administered 1.0 × 10^8^ CFUs of Rh-YBS on the 8_th_ day and YBS on the 10_th_, 12_th_, and 14_th_ days; the Rh-YBS-2 group received 1.0 × 10^8^ CFUs of Rh-YBS on the 8_th_ and 10_th_ days, and YBS on the 12_th_ and 14_th_ days; the Rh-YBS-3 group was administered 1.0 × 10^8^ CFUs of Rh-YBS on the 8^th^, 10_th_, and 12_th_ days, and YBS on the 14_th_ day. The dosage of Rh was 32 μg, and for YBS and Rh-YBS, it was 1.0 × 10^8^ CFUs. A control group without DSS treatment was orally administered PBS on the 8_th_, 10_th_, 12_th_, and 14_th_ days. The Disease Activity Index (DAI) was recorded and the mice were sacrificed on the 15_th_ day. Colons, serum, feces, and primary organs (heart, liver, spleen, lung, and kidney) were collected for further experimentation.

*Evaluation of* *prevention efficacy of Rh-YBS for UC in vivo*

Mice were randomly divided into five groups and given 3% DSS in deionized water for seven days. Concurrently, mice were orally administered 0.2 mL PBS (Model group), 0.2 mL BS (BS group), 0.2 mL YBS (YBS group), 0.2 mL 0.1% Lam + 0.2 mL Rh-YBS (Lam + Rh-YBS group), and 0.2 mL Rh-YBS (Rh-YBS group) every 2 days. Additionally, a control group was given deionized water and orally administered 0.2 mL PBS every two days. The health statuses of the mice were monitored and they were sacrificed on the 10_th_ day. Colons, serum, feces, and primary organs (heart, liver, spleen, lung, and kidney) were collected for further experimentation.

*Statistical analysis*

This study employed a random sampling method and the data were analyzed using the analysis of variance (ANOVA) and the least significant difference test among groups (n ≥ 3). All values were presented as the mean ± standard error (SE). Differences were considered statistically significant at **p* < 0.05, ** *p* < 0.01.

**Reference**

[1] P. Xiulan, Y. Naijing, L. Meisi, C. Qiyan, D. Lingling, X. Haiting, L. Rui, H. Xiaoqin, Q. Shanshan, N. Wenbiao, H. Haoqi, W. Yanli, D. Linxin, L. Dasheng, G. Fei, *Carbohydrate Polymers* **2021,** *273*, 118612.

[2] C. Qiyan, L. Rui, H. Xiaoqin, Z. Jinming, H. Yao, Q. Shanshan, P. Xiulan, N. Wenbiao, D. Lingling, X. Haiting, L. Fang, L. Meisi, Z. Heng, F. Chaomei, G. Fei, *Biomacromolecules* **2021,** *22*, 2754.

[3] S. Feng, L. Mengmeng, Z. Di, L. Xianhe, Z. Lei, W. Zhe, G. Ning-ning, *Chinese Herbal Medicines* **2017,** *9*, 388.

[4] Z. Hong, F. Jing, W. Hongwei, Y. Hongjun, L. Hua, W. Zhuju, Y. Bin, T. Liying, F. Meihong, *Chinese Herbal Medicines* **2017**, 10.15212/amm-2023-0044.

[5] C. Qiwen, Q. Jiyan, C. Mengwei, H. Ziyi, Z. Xuan, Z. Xianzheng, *Materials Today* **2023,** *63*, 32.

[6] L. Sisi, M. Subhajit, L. Juanjuan, H. Weiliang, P. Chao, L. Jinyao, *Science Advances* **2021,** *7*, 20.

[7] L. Rui, L. Meisi, Z. Chen, S. Jinfeng, Z. Siyuan, C. Qiyan, H. Yichen, Z. Minyue, Z. Jinming, G. Fei, *Food Chemistry* **2020,** *330*, 127241.

[8] L. Ruifeng, L. Meisi, F. Chaomei, Z. Jinming, C. Qiyan, Z. Chen, S. Jinfeng, P. Xiulan, D. Lixin, X. Haiting, Y. Naijing, S. Jiayi, L. Dasheng, D. Bin, M. Arlene, F. Shu Ling, G. Fei, *Carbohydrate Polymers* **2021,** *263*, 117998.

[9] W. Marta, A.T. Christoph, N. Roni, H.M. Jorge, Z. Jianping, B. Eric, F. Gad, L. Maayan, N.K. Mark, M.P. William, E. Eran, B.B. Finlay, A.F. Richard, *Cell* **2014,** *156*, 1045.

[10] W. Yanli, Z. Shaojin, Y. Ke, L. Rui, D. Linxin, Z. Wang, Y. Yonghong, F. Chaomei, L. Dasheng, L. Nan, C. Jianping, Z. Chuan, F. Shu Ling, G. Fei, *International Journal of Biological Macromolecules* 2023, *260*, 128818.

[11] D. Lingling, D. Hongling, Z. Minyue, X. Haiting, P. Xiulan, C. Qiyan, L. Rui, H. Yichen, W. Ying, T. He, Z. Jinming, G. Fei, *Phytotherapy Research* **2022,** *36*, 2081.

**Table and Figures**

**Table S1.** Detection of Rh by HPLC. Data are mean ± SE (n=4).

| Rh concentration (μg/mL) | Peak area |
| --- | --- |
| 384 | 8749633 |
| 192 | 4179634 |
| 96 | 1982318 |
| 48 | 925536 |
| 24 | 422162 |
| 12 | 197513 |
| 6 | 85768 |
| 3 | 44353 |
| Sample | 1460841 ± 158852 |


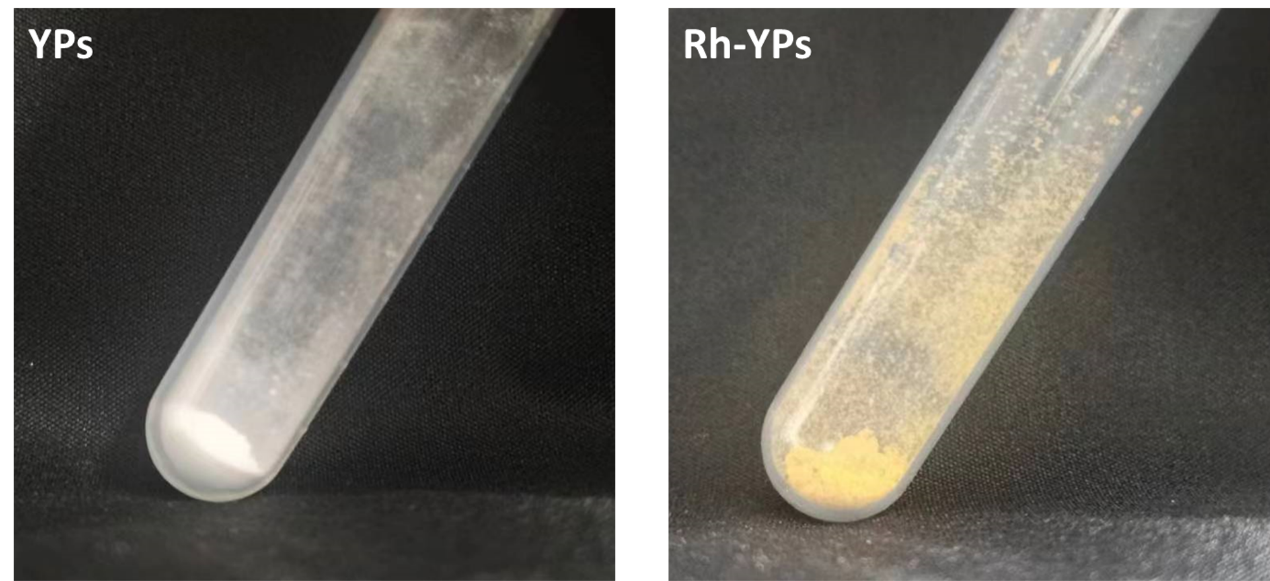


**Figure S1.** Appearance of YPS and Rh-YPS


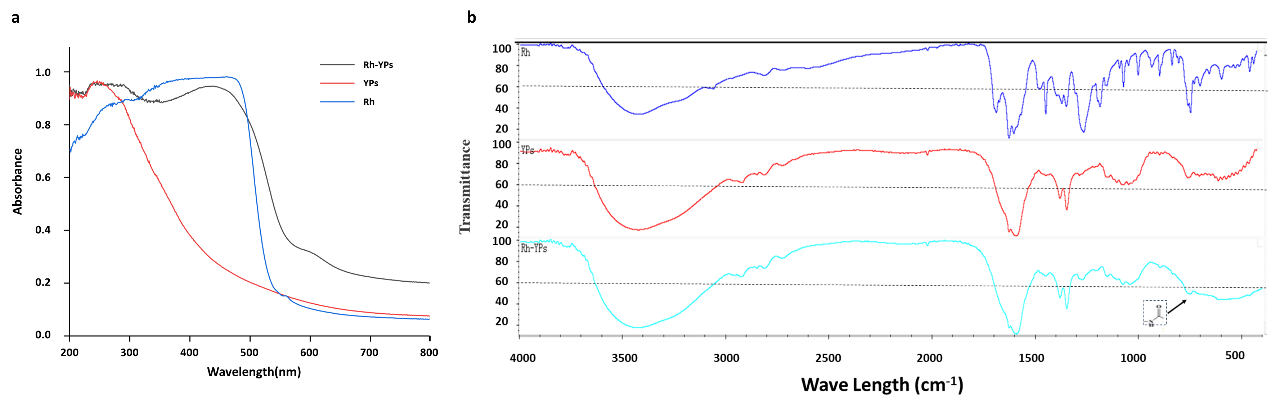


**Figure S2.** Characterization of Rh-YPs. a) UV spectra of Rh, YPs and Rh-YPs. b) IR spectra of Rh, YPs and Rh-YPs.


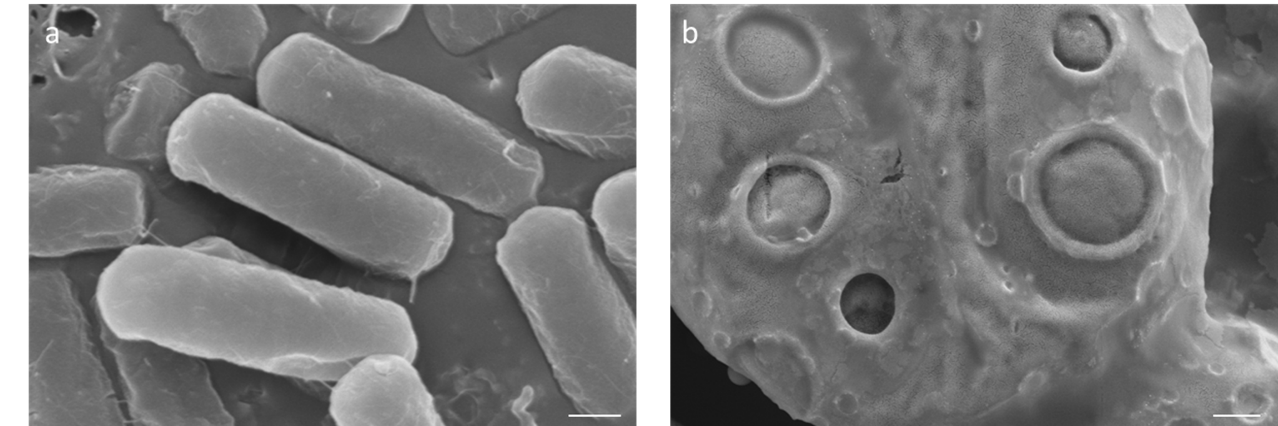


**Figure S3.** SEM image of a) BS and b) Rh-YPs Scale bar: 1 μm.


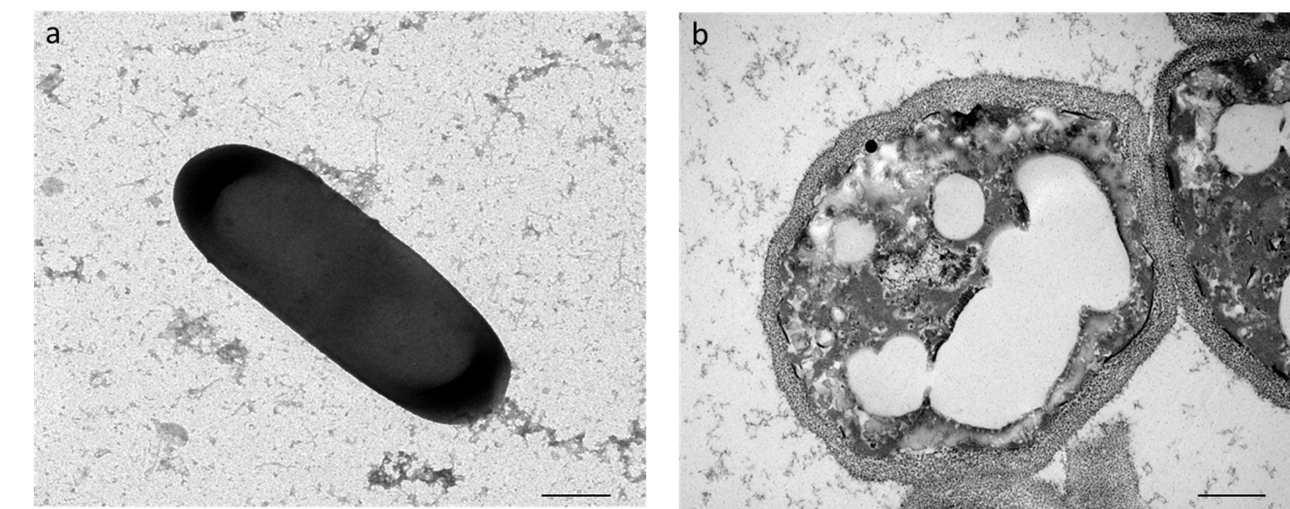


**Figure S4.** TEM image of a) BS and b) Rh-YPs. Scale bar: 1 μm.


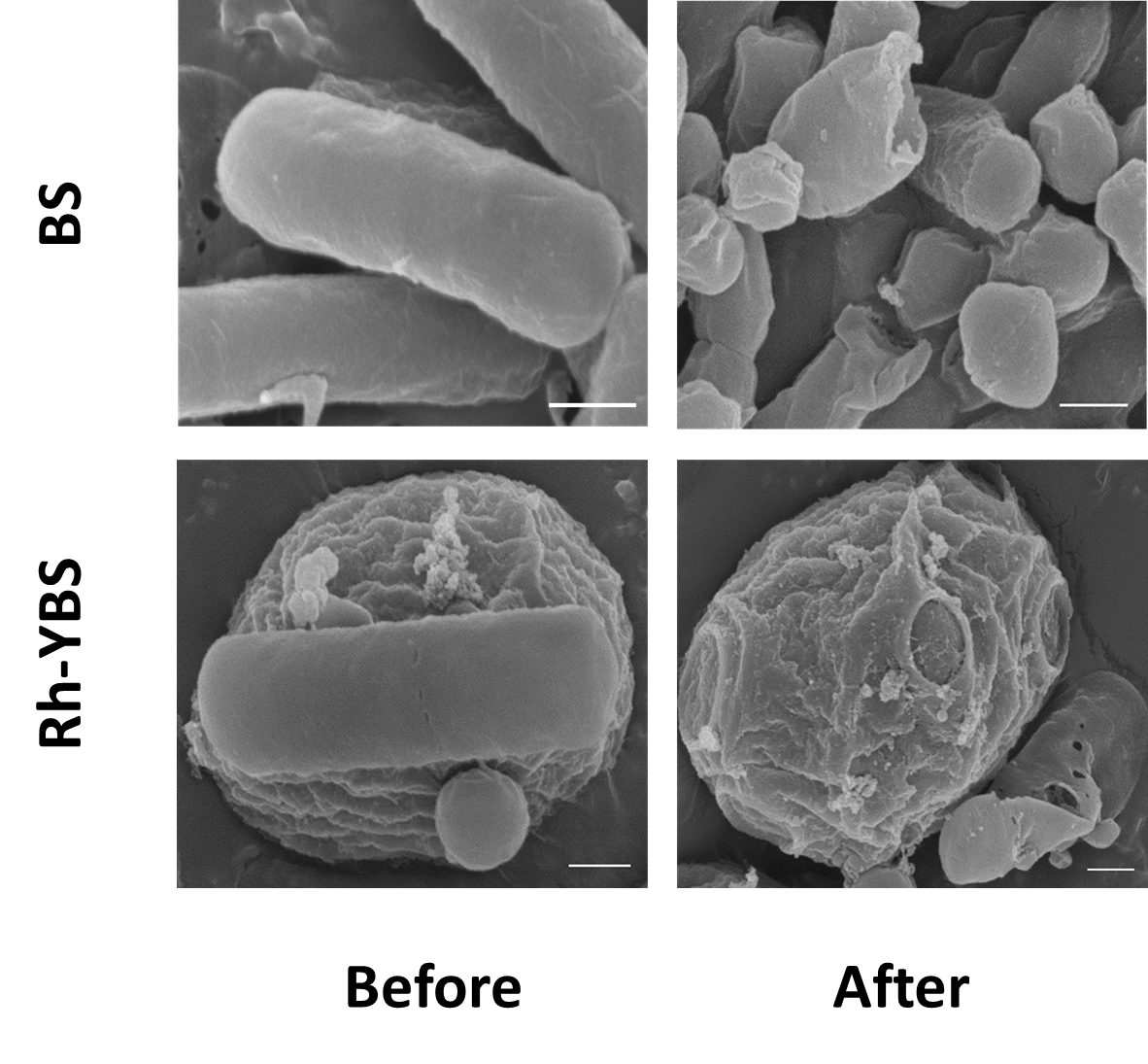


**Figure S5.** SEM image of BS and Rh-YPs before and after treatment of SGF. Scale bar: 1 μm.


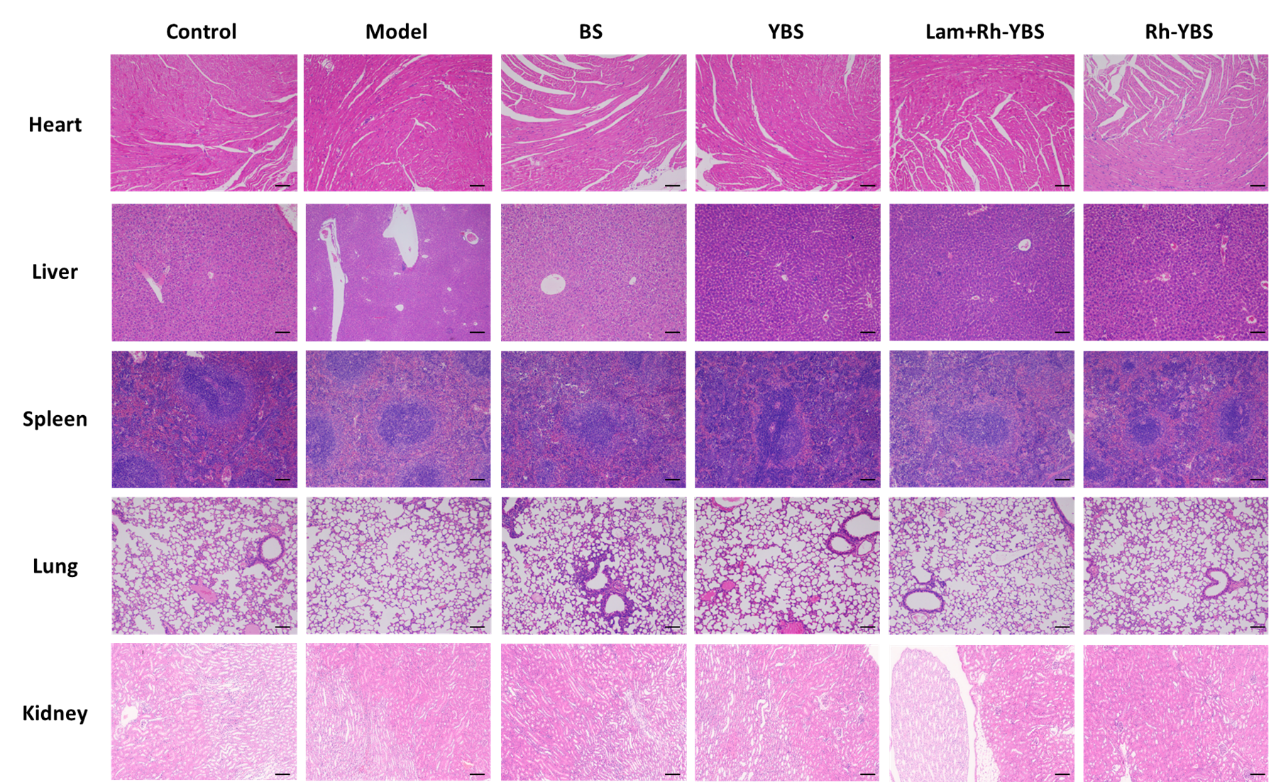


**Figure S6.** H&E stain of mean organs of mice from **Basic effects of Rh-YBS *in vivo***. Scale bar: 100 μm.


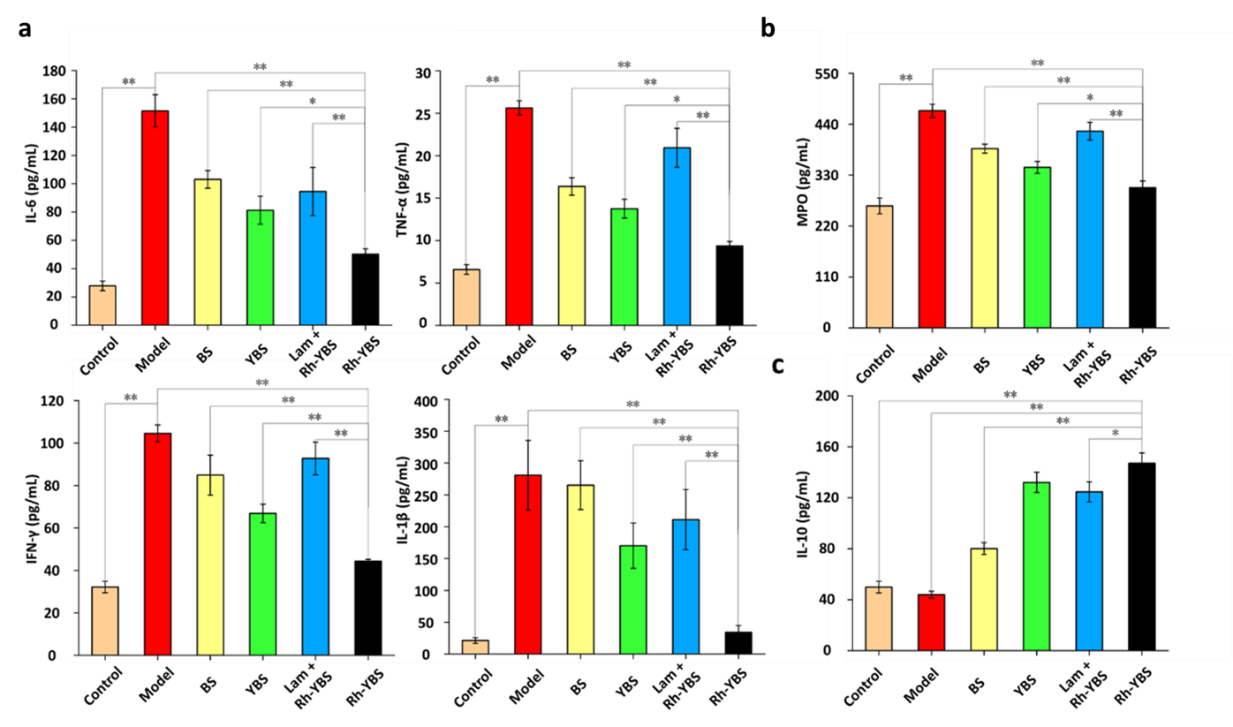


**Figure S7.** Histogram analysis of different groups on the changes of a) inflammatory cytokines (IL-6, TNF-α, IFN-γ, and IL-1β), b) oxidation cytokines (MPO), c) anti-inflammatory cytokines (IL-10) in the serum from **Basic effects of Rh-YBS *in vivo*** (n=6). Data are mean ± SE. **p* < 0.05; ***p* < 0.01.


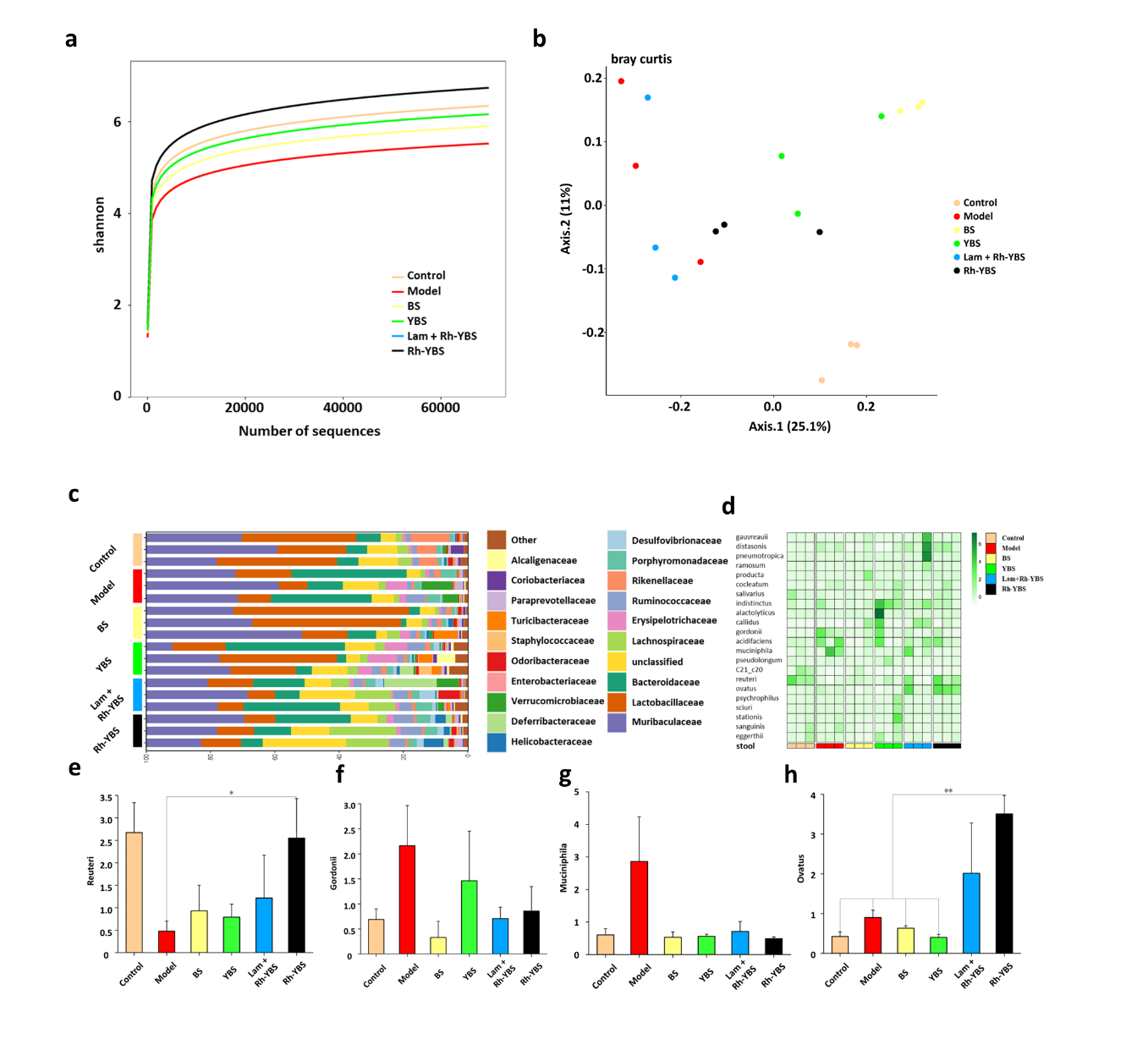


**Figure S8.** Analysis of fecal flora of mice from **Basic effects of Rh-YBS *in vivo***. a) Alphadiversity of each group. b) Beta diversity of each group. c) Family-level square stacking diagram d) Species-level clustering heat map. e)-h) Relative abundance level of *Reteri*, *Muciniphila*, *Gordonii*, and *Ovatus*.


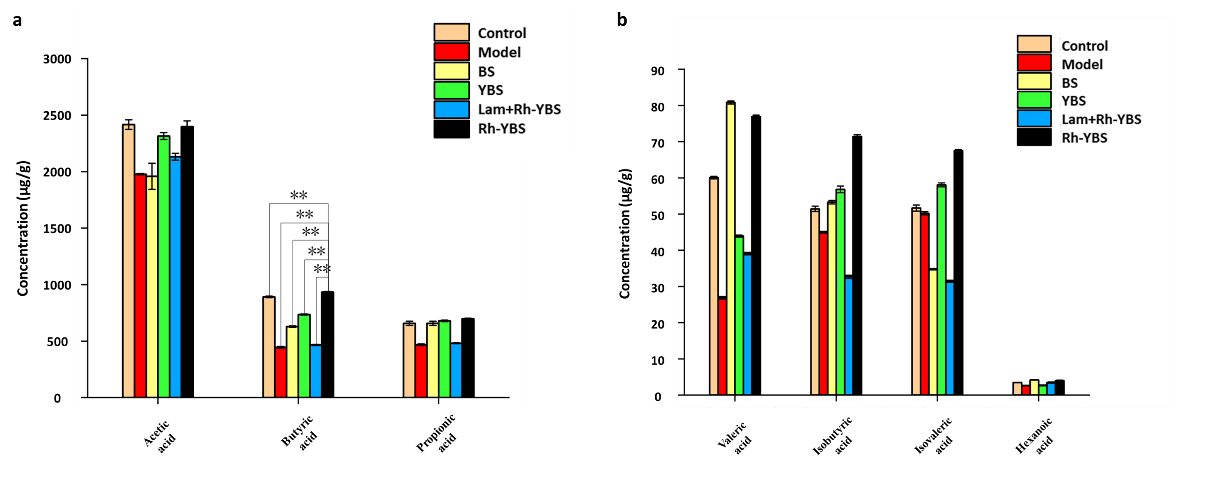


**Figure S9.** SCAFs content in feces of mice from **Basic effects of Rh-YBS *in vivo*** (n=5). Data are mean ± SE. **p* < 0.05; ***p* < 0.01.


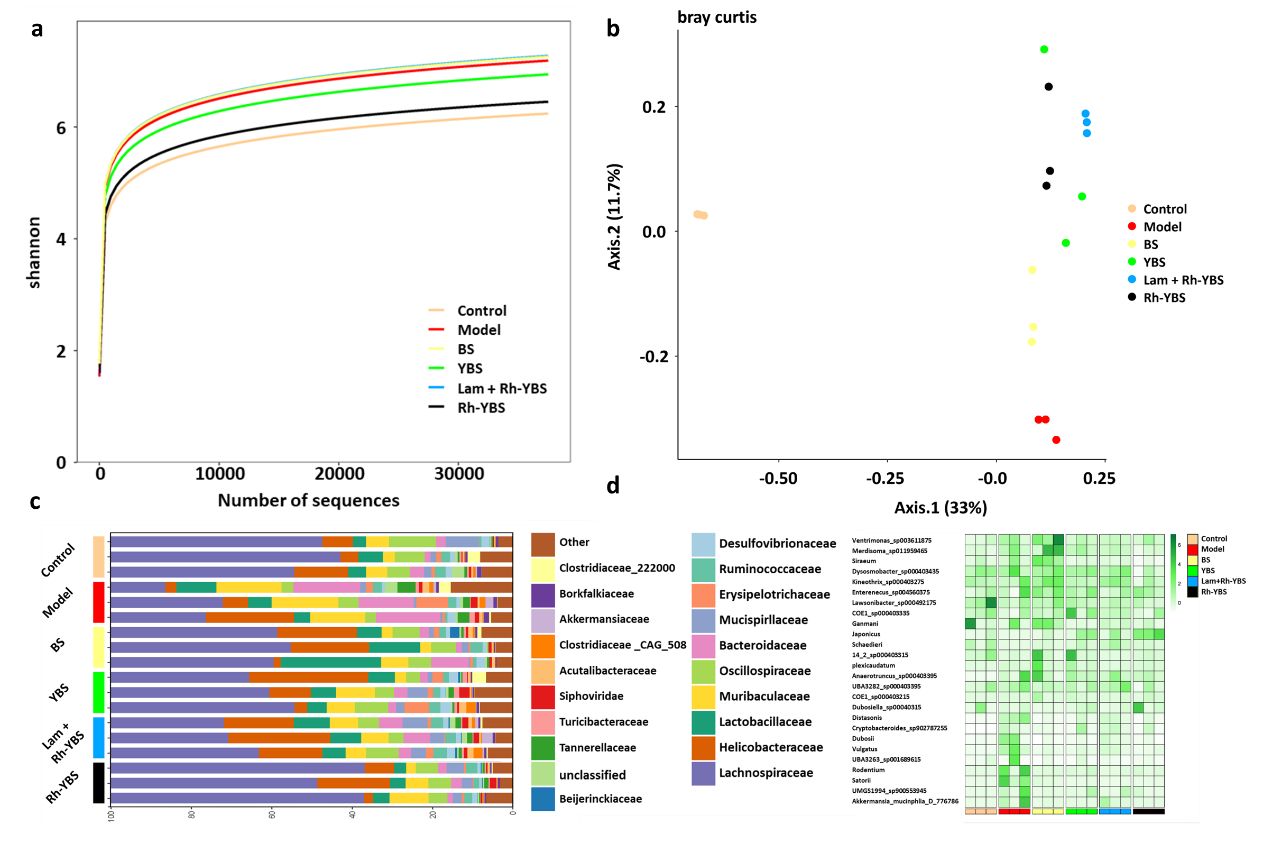


**Figure S10.** Analysis of submucosal flora of mice from **Basic effects of Rh-YBS *in vivo***. a) Alphadiversity of each group. b) Beta diversity of each group. c) Family-level square stacking diagram d) Species-level clustering heat map.


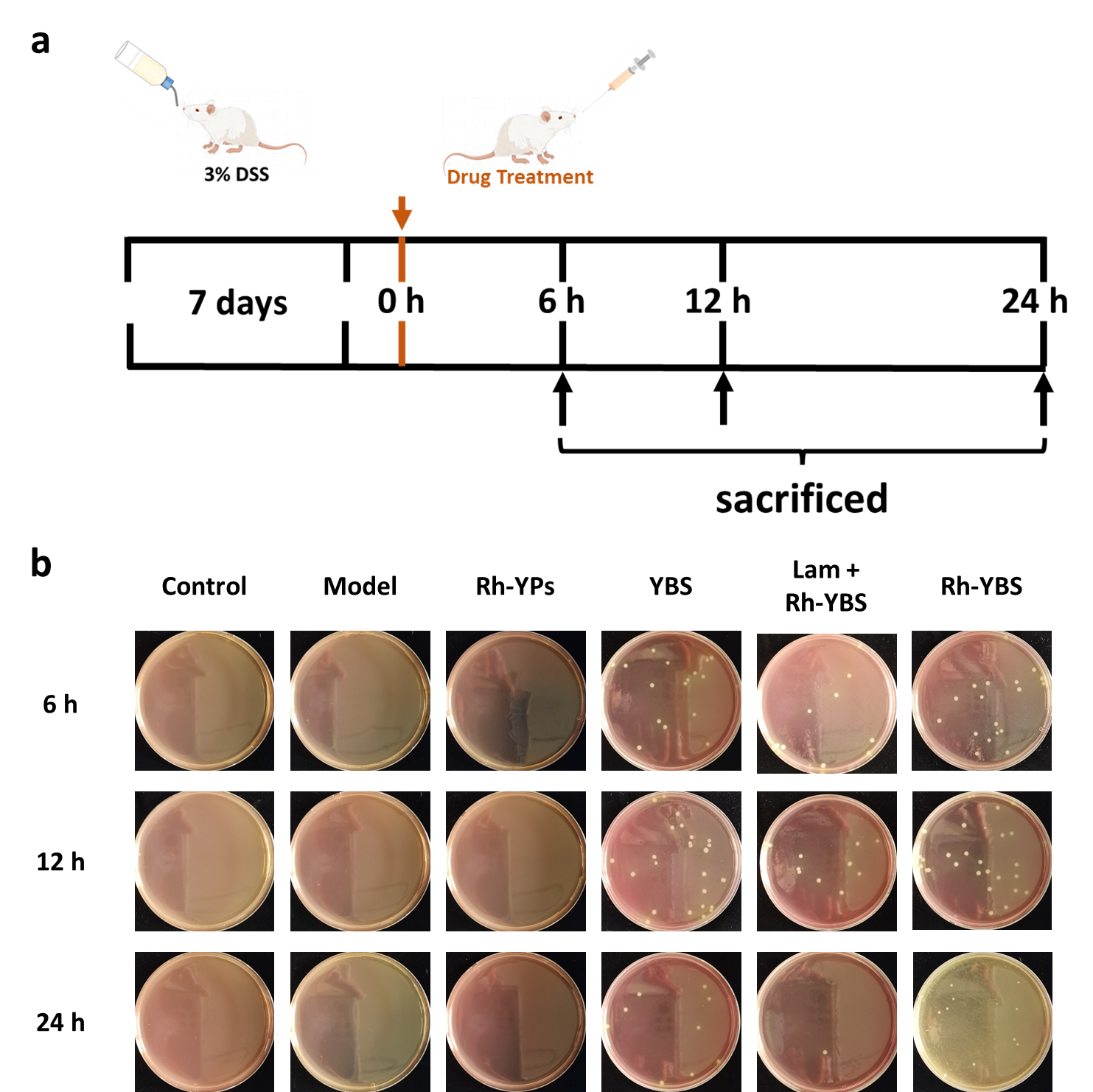


**Figure S11.** Contributions of BS to Rh-YBS. a) Therapeutic procedure of different therapeutic formulations to ABX mice. b) The colonize counts of BS from submucosa of ABX mice.


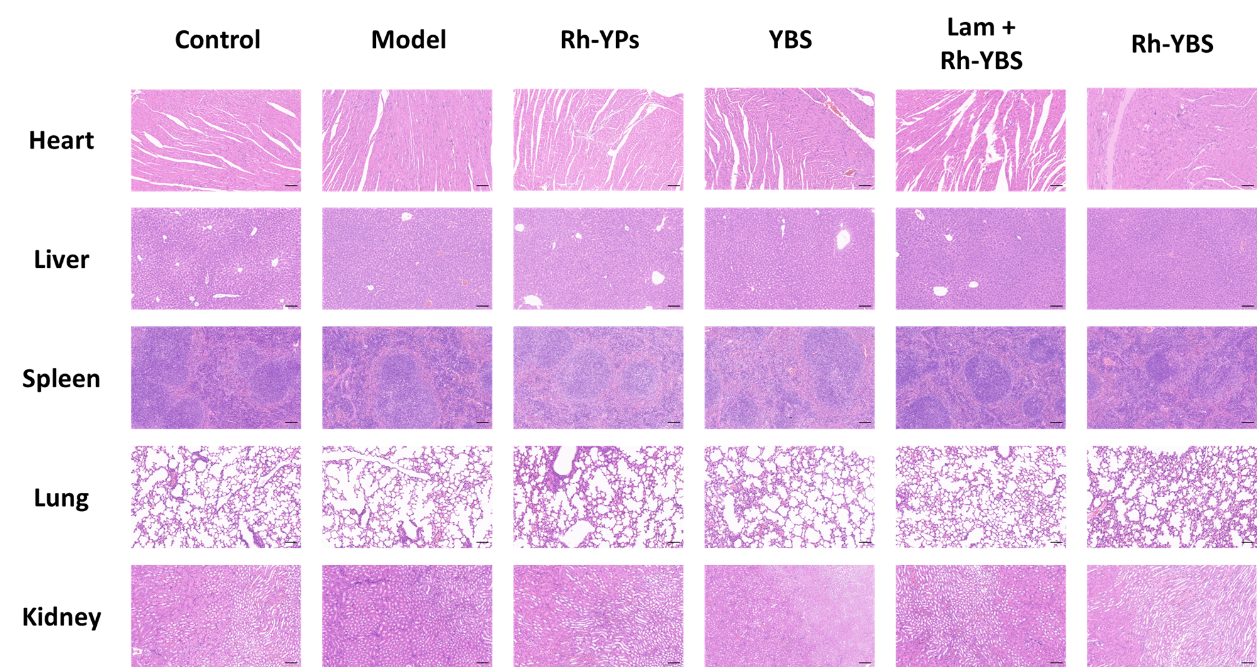


**Figure S12.** H&E stain of mean organs of mice from **Contributions of BS to Rh-YBS.** Scale bar: 100 μm.


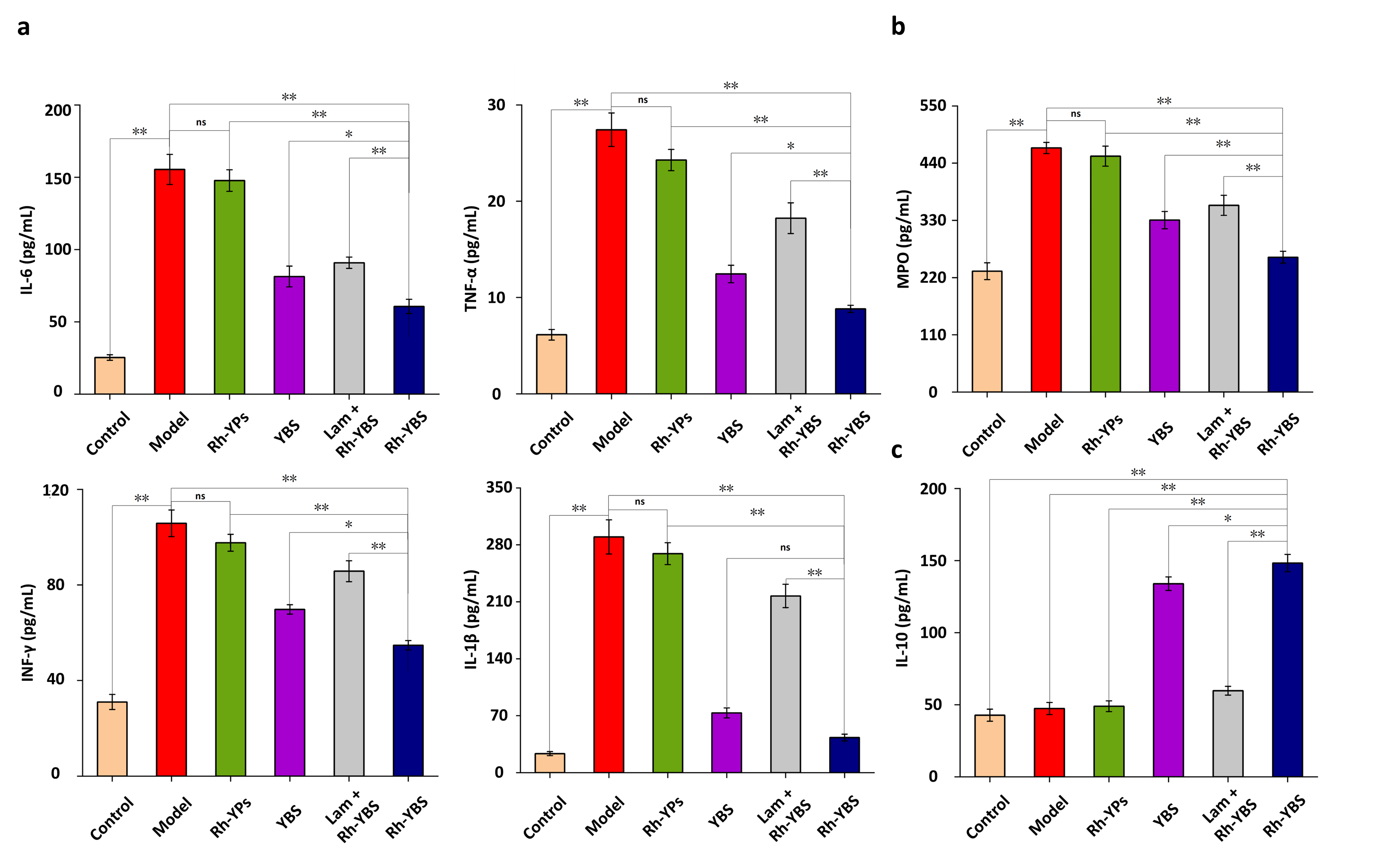


**Figure S13.** Histogram analysis of different groups on the changes of a) inflammatory cytokines (IL-6, TNF-α, IFN-γ, and IL-1β), b) oxidation cytokines (MPO), c) anti-inflammatory cytokines (IL-10) in the serum from **Contributions of BS to Rh-YBS** (n=6). Data are mean ± SE. **p* < 0.05; ***p* < 0.01.


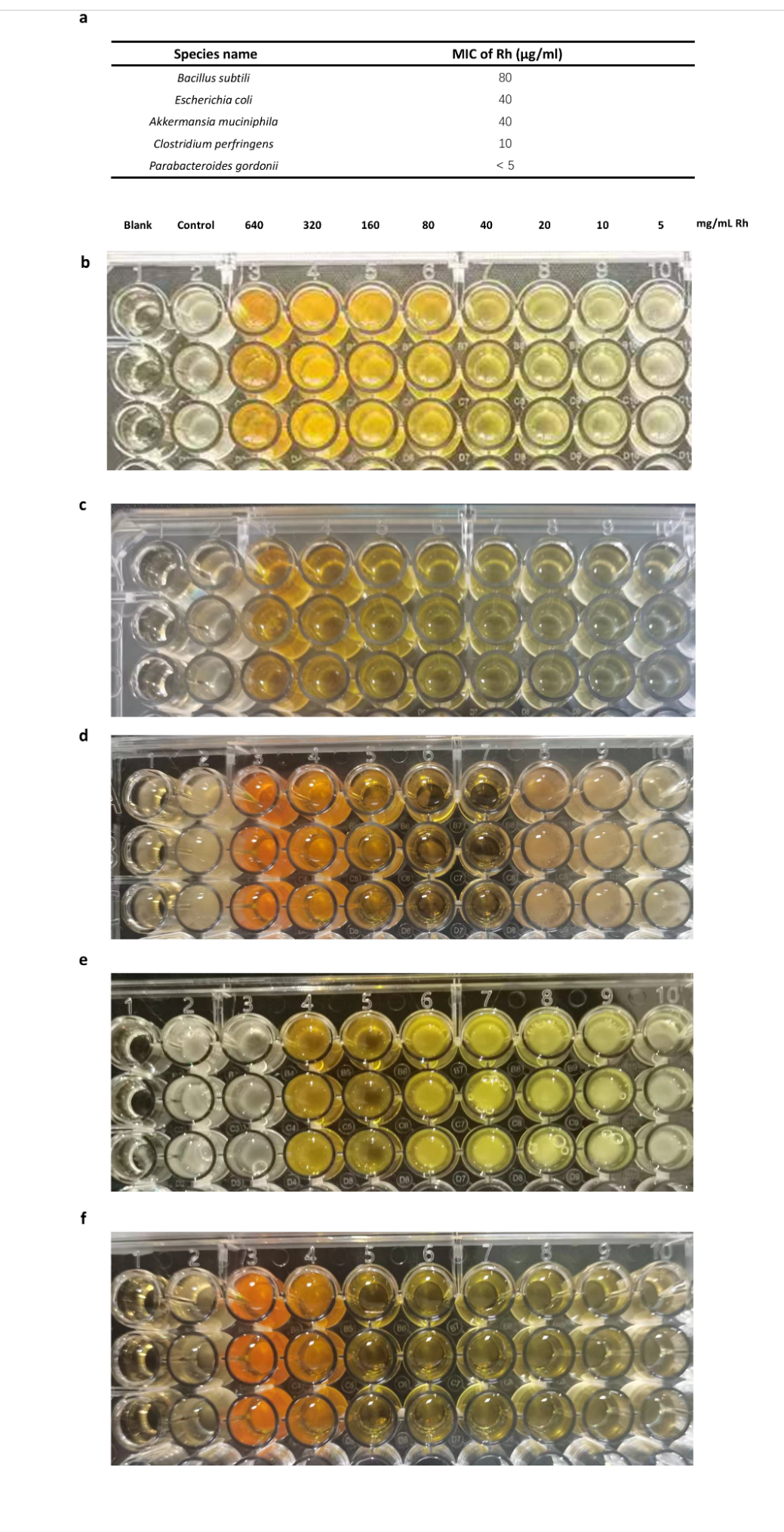


**Figure S14.** Antibacterial ability of Rh to b) BS. c) *E. coli.* d) *Akkermansia muciniphila*. e) *Clostridium perfringens.* f) *Parabacteroides gordonii.*


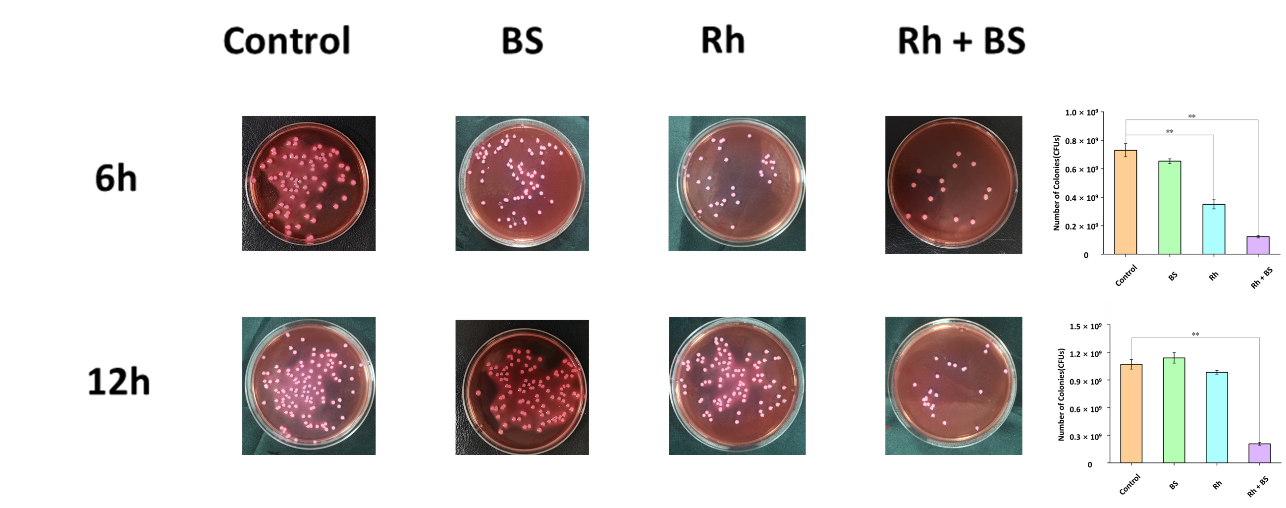


**Figure S15.** Rh assisted BS against *E. coli*.


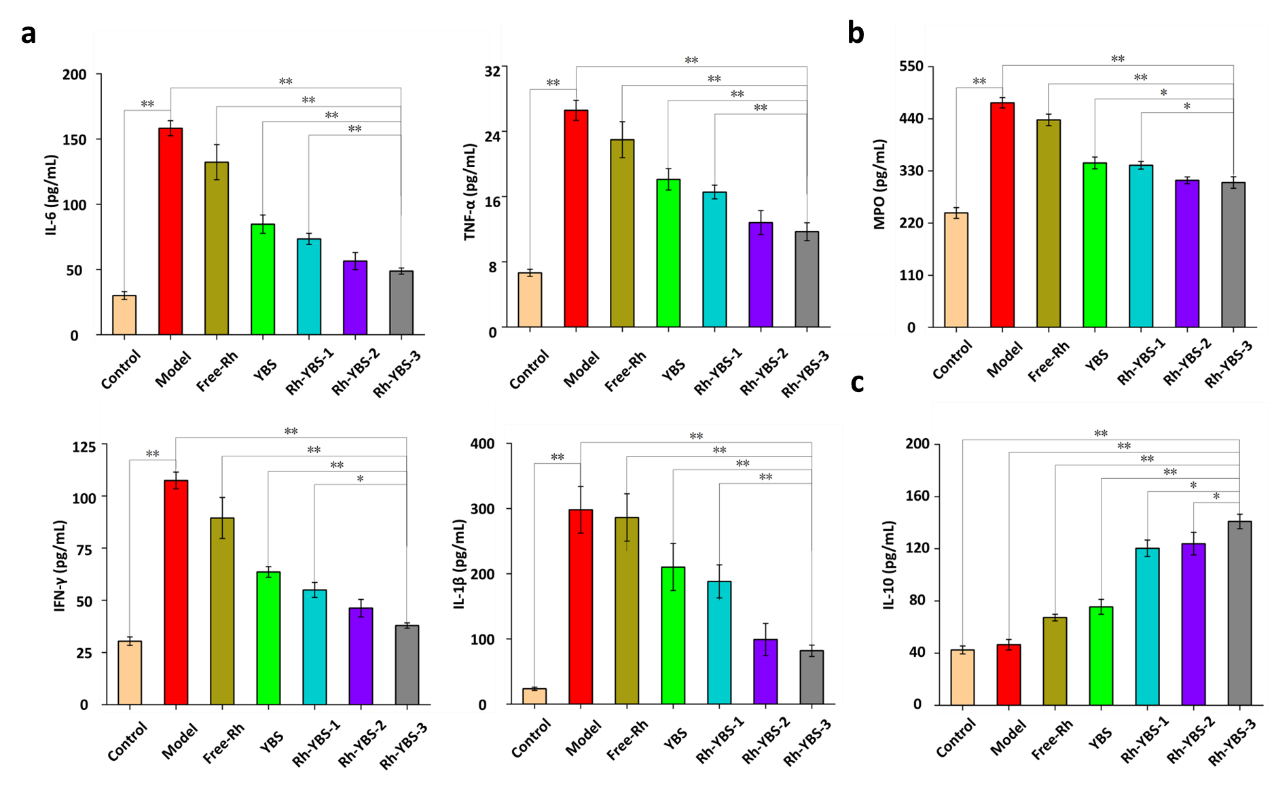


**Figure S16.** Histogram analysis of different groups on the changes of a) inflammatory cytokines (IL-6, TNF-α, IFN-γ, and IL-1β), b) oxidation cytokines (MPO), c) anti-inflammatory cytokines (IL-10) in the serum from **Contributions of Rh to Rh-YBS** (n=6). Data are mean ± SE. **p* < 0.05; ***p* < 0.01.


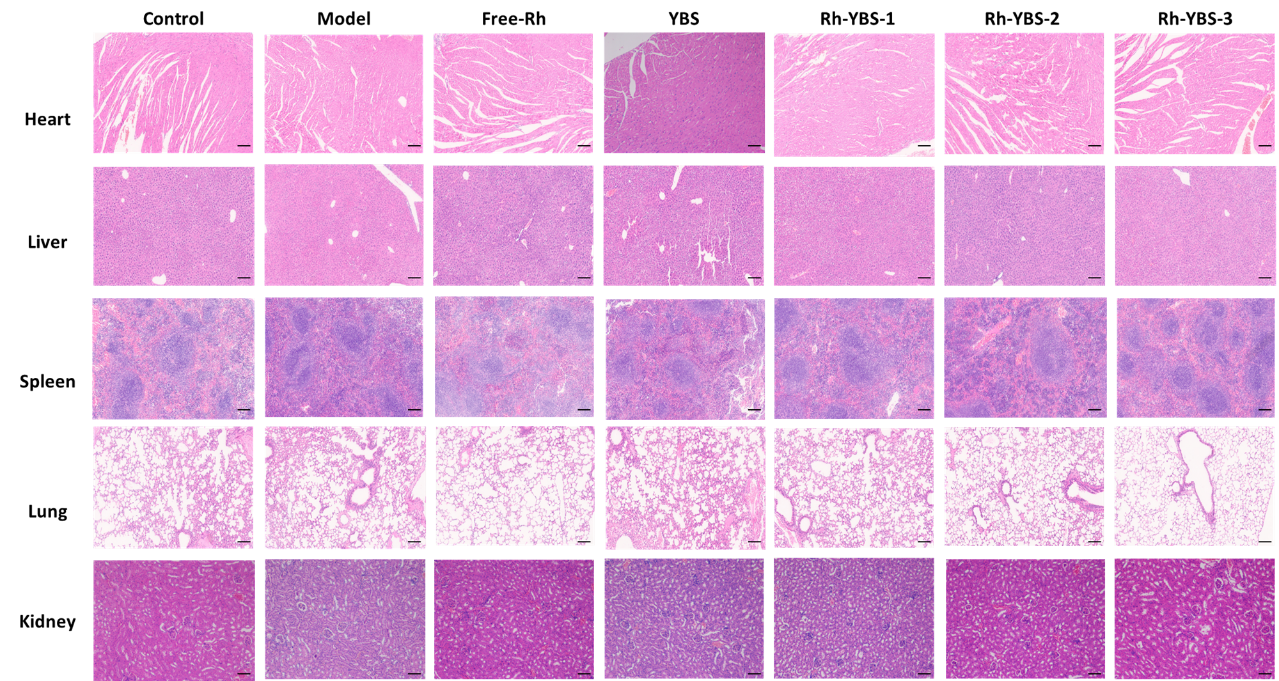


**Figure S17.** H&E stain of mean organs of mice from **Contributions of YPs to Rh-YBS.** Scale bar: 100 μm.


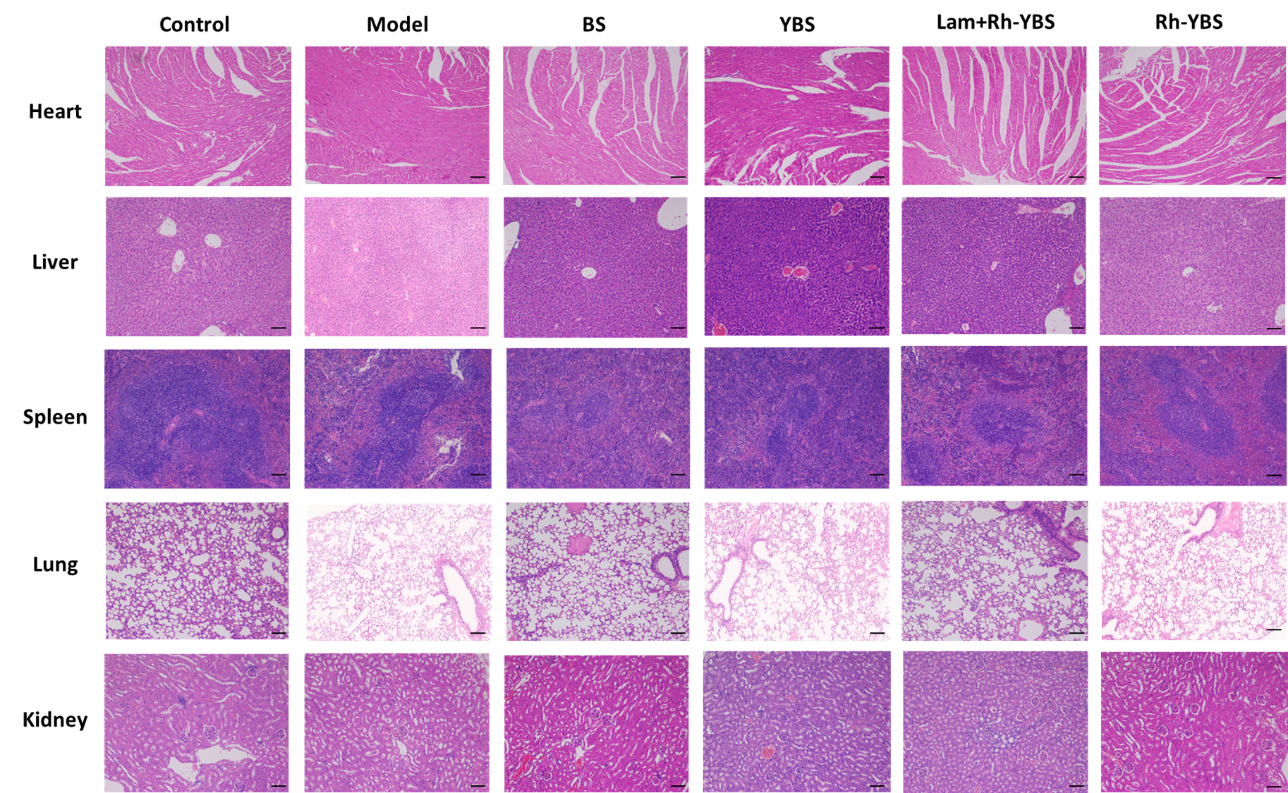


**Figure S18.** H&E stain of mean organs of mice from Evaluation of the **Preventive efficacy of Rh-YBS in UC**. Scale bar: 100 μm.


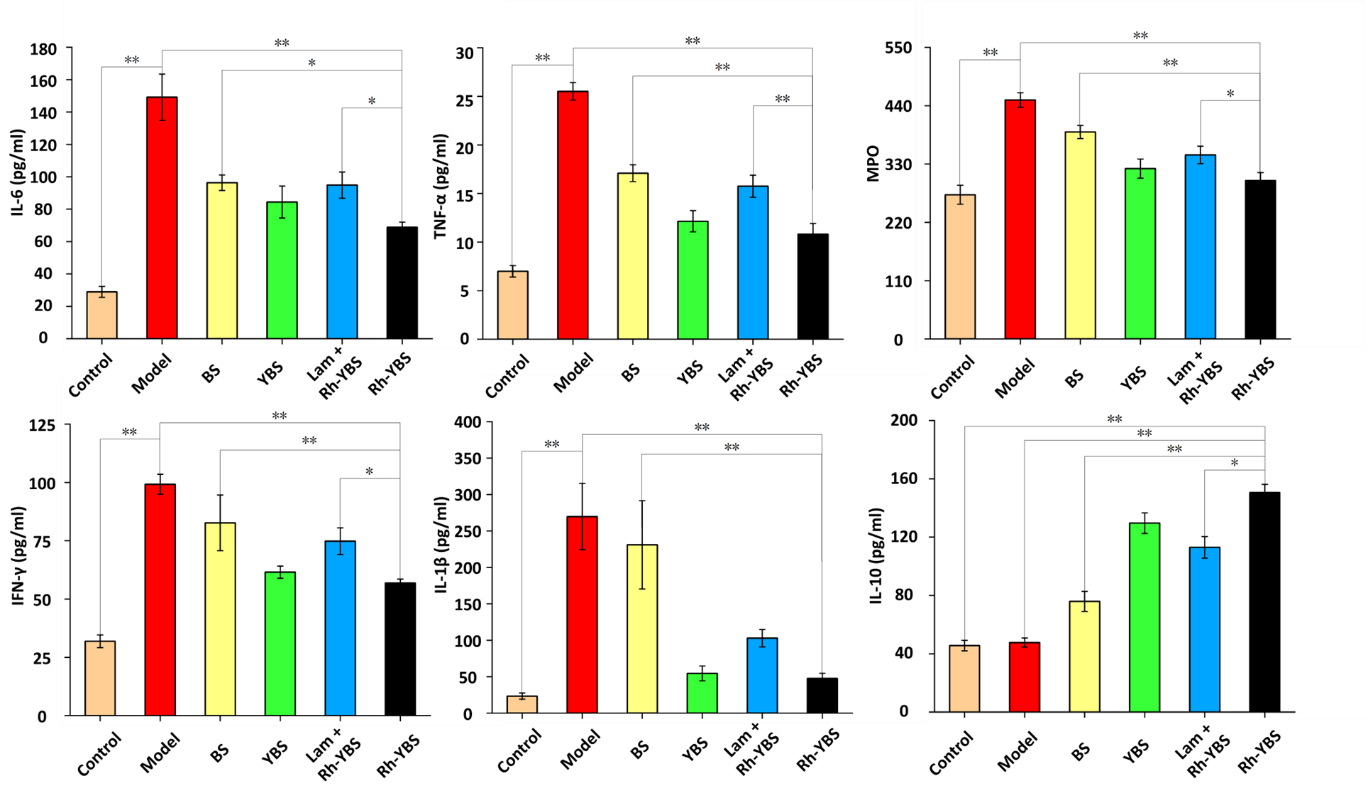


**Figure S19.** Histogram analysis of different groups on the changes of a) inflammatory cytokines (IL-6, TNF-α, IFN-γ, and IL-1β), b) oxidation cytokines (MPO), c) anti-inflammatory cytokines (IL-10) in the serum from **Evaluation of the Preventive efficacy of Rh-YBS in UC** (n=6). Data are mean ± SE. **p* < 0.05; ***p* < 0.01.
